# High-Resolution Mass Spectrometer–Based Ultra-Deep Profile of Milk Whey Proteome in Indian Zebu (*Sahiwal*) Cattle

**DOI:** 10.3389/fnut.2020.00150

**Published:** 2020-09-11

**Authors:** Alka Chopra, Syed Azmal Ali, Shveta Bathla, Preeti Rawat, Vikas Vohra, Sudarshan Kumar, Ashok Kumar Mohanty

**Affiliations:** ^1^Proteomics and Cell Biology Lab, Animal Biotechnology Center, National Dairy Research Institute, Karnal, India; ^2^Yale University School of Medicine, New Haven, CT, United States; ^3^Animal Genetics and Breeding Division, National Dairy Research Institute, Karnal, India

**Keywords:** host defense, Indian zebu, mass spectrometer, milk whey, proteomics, *Sahiwal*

## Abstract

Milk serves as a mode of protection to neonate through transferring the host defense proteins from mother to offspring. It also guards the mammary gland against various types of infections. Along with the presence of six vital proteins, bovine milk (whey) contains a massive class of minor proteins, not all of which have been comprehensively reported. In this study, we performed an LC-MS/MS-based ultra-deep identification of the milk whey proteome of Indian zebu (*Sahiwal*) cattle. Three independent search engines that are Comet, Tandem, and Mascot-based analysis resulted in the discovery of over 6,210 non-redundant proteins commonly identified. Genome-wise mapping revealed that chromosome 1 showed a minimum expression of 14 proteins, whereas chromosome 19 expressed 250 maximum proteins in milk whey. These results demonstrate that milk proteome in *Sahiwal* cattle is quite complicated, and minor milk fractions play a significant role in host defense.

## Introduction

Milk synthesis is an absolute feature that defines the mammalian class. It has received considerable interest because of its nutritional and functional properties to newborn and young offspring (1). It majorly acts as a vital source for the transfer of defense molecules against pathogens from mother to child. Worldwide, humans consume bovine milk in their diet, and a regular diet accounts for 80–90% of total bovine milk intake and acts as an essential source of nutrition to them. Milk acts as a medium for the transfer of host defense proteins, yet its repertoire of minor proteins has been only partly characterized (2). In addition, bovine milk is exceedingly being utilized for dairy products, including yogurt, curd, cheese, butter, and, to some extent, as bioactive peptides (3).

Milk from different species was previously studied as a substitute for human milk in infant food. Several studies were mainly focused on major milk components (4). The low abundant proteins in milk could be explored with the development of analytical methods. The comprehensive understanding of milk of different species could be beneficial in promoting the utilization of milk as a source of nutrition. Attaining a complete knowledge of the milk whey proteome could serve as a significant foundation for the production of functional foods and infant products.

More recently, proteomic approaches based on mass spectrometry have been used for farm animal milk proteome research (5). Various proteomic approaches have been identified for exploring the molecular pathways and cellular functions of the complex milk proteins in caseins, whey protein, and milk-fat globular membrane (6). Proteomic studies have been conducted for exploring the milk whey proteome in humans and bovine for the identification of proteins related to host defense and immune system (7).

*Sahiwal* (*Bos indicus*) cattle breed is the dominant milch breed of Indian origin having native tract in North-Western region of India but a much broader breeding tract in the country. In terms of milk production, it is a high milk-producing breed (8); it is known to be genetically more thermotolerant and less disease susceptible (9). In the tropics, *Indicine* cattle have lesser disease susceptibility for reproductive and productive disorders and parasitic infections compared to *taurine* cattle (10). Also, Indian native zebu cattle (*Sahiwal*) can survive comparatively well on low input production systems with moderate milk production but yield much more when maintained on the intensive system of production. Therefore, it is crucial to generate the proteome profile of Indian zebu cattle with the aim of identifying low abundant proteins involved in the host defense mechanism of *Indicine* cattle. The protein composition of milk varies with genetic and non-genetic factors (11, 12). In cow milk, the significant determinants of milk proteome include breed, feed conversion, stage of lactation, parity of production, and environmental determinants (13). The foremost fraction of milk includes caseins, and ~20% of milk composition is the whey proteome comprising alpha-lactalbumin and beta-lactoglobulin. Bioactive proteins and peptides in cow milk were reported to play a different role in cellular and physiological functions. It acts as a significant determinant in the development of immune response, protective functions against fungal, bacterial, and viral infections, and milk also plays a vital role in the maintenance of intestinal microbiota (14). Most of the reported proteins responsible for high biological activity are immunoglobulins, lactoferrin, α-lactalbumin, β-lactoglobulin, αS1-, αS2-, β-, k-caseins, and lactadherin (15). The presence of immunoglobulins in whey confers the initial line of passive defense to neonates, and in adults, it regulates a person's immune system (16). β-Lactoglobulin and lactoferrin fractions of milk proteome exhibit anticarcinogenic, immunomodulatory, antimicrobial, and antioxidant responses (17). Therefore, it is essential to perform the in-depth comparison of whey proteome of different species for unraveling the diversity in genetics and biological traits for a given species. Previously, a study identified the limited number of 211 proteins but demonstrated the uniqueness of a few proteins in the given species (1). Recent reports suggest the comparison of milk proteins within Holstein and Jersey breeds of dairy cattle (18). They reported differences in the low abundant proteins present in the skimmed milk fractions of Holstein and Jersey breeds fed on the same diet with similar management conditions. A report on comparison of different lactation stage proteins was conducted in Malanad Gidda (*Bos indicus*) cattle (19); however, to date, no such report is available in Indian zebu (*Sahiwal*) cattle. The present study was conducted to explore the milk whey proteome of Indian zebu cattle. As per our knowledge, it is the first report of milk whey proteome of *Sahiwal* cattle of Indian origin and can be used as a comprehensive reference database for cattle genetic resources of India.

## Materials and Methods

### Chemicals and Reagents

All chemicals, reagents, and organic solvents were purchased from Sigma-Aldrich (St. Louis, MO, USA) unless otherwise specified. Ultra-pure grade water was used throughout. All reagents used for this study were of molecular grade.

### Selection of Animals for Milk Collection

Indian zebu (*Bos indicus*) cattle, *Sahiwal* breed (*n* = 10), in different significant lactation phases were used for the current study. The animals were reared in the animal herd under uniform feeding and breeding practices at the ICAR–National Dairy Research Institute, Karnal, India. Before the study, all animals were screened for the absence of mastitis by the California mastitis test. Only the healthy cows with somatic cell count ≤200 lakhs per cell without any sign of mammary gland infection were included in the study, and it was ascertained that the milk is free from bacteria. Before the collection of milk, the rectal examination for the absence of high body temperature was obtained twice a day, and cows having normal body temperature range were used for milk collection. All cows had free access to water and offered the same *ad libitum* diet.

The ICAR–National Dairy Research Institute, Karnal, India, animal care, and the committee approved all procedures used in this study. The milk samples were immediately transported to the laboratory under an icebox within 1 h and pooled before the experiment. The pooled samples aim to reduce the animal-to-animal variations.

### Sample Preparation

Raw milk samples were defatted by centrifugation at 4,000 rpm for 15 min to obtain skimmed milk samples. Subsequently, the skimmed milk samples were ultracentrifuged at 65,000×*g* for 2 h at 4°C for separating casein, and the liquid portion was referred to as milk whey. Phenyl methyl sulfonate was added at a concentration of 0.01% to prevent proteolytic degradation of milk whey samples. The samples were stored at −80°C for further experimentation and analysis.

### Optimization by Various Protein Extraction Methods

Various protein extraction procedures were followed and optimized, which includes (a) ultracentrifugation followed by acetone precipitation, (b) CaCl_2_ precipitation methods at different concentrations (60, 90, and 120 mM), and (c) TCA/acetone precipitation methods, respectively.

- **Ultracentrifugation followed by acetone precipitation**
  The milk was subjected to ultracentrifugation at 65,000×*g* for 2 h for whey preparation with minor modifications (7). Whey samples were further precipitated by adding chilled acetone and incubated at −20°C for 14–16 h followed by protein precipitation at 13,000 rpm at 4°C for 10 min (20). The supernatant was discarded, and the pellet was reconstituted with 1× PBS by adding 0.2% DEA (diethylamine) and 20% 0.5 M Tris of pH 6.8. Subsequently, the sample was dried in the vacuum-sealed concentrator and processed for tryptic digestion and mass spectrometric analysis.
- **CaCl**_**2**_ **precipitation**
  CaCl_2_ precipitation method was optimized at different concentrations (60, 90, and 120 mM) of CaCl_2_, respectively, for precipitating milk whey proteins from defatted milk samples followed by SDS-PAGE profiling of the whey precipitate.
- **TCA/acetone precipitation**
  TCA/acetone precipitation method was used for precipitating protein samples. Then 1.5 ml of 10% TCA/acetone was added to the whey sample and stored at −20°C overnight (21). The sample was centrifuged at 13,000 rpm for 10 min at 4°C, and the supernatant was discarded. The pellet was further re-suspended in 1× PBS and 2% DEA (diethylamine) for pellet dissolution.

### Protein Quantitation

2D-Clean Up kit (GE Healthcare, USA) was used for removal of interfering substances from precipitated milk protein preparation, and total protein concentration was estimated using Bradford protein estimation kit (Bio-Rad) as per the manufacturer's instruction.

### SDS-PAGE

Twenty-five micrograms (25 μg) of pooled Proteo-miner enriched milk whey sample (pooled from 10 animals of different lactation stages, *n* = 10), respectively, were subjected to 12% SDS-PAGE (10 × 10.5 cm) in a Mini VE complete gel electrophoresis system (GE Healthcare, USA). The gel was stained with colloidal Coomassie brilliant blue dye for in-gel digestion.

### *In-gel* Tryptic Digestion

The gel lane with the enriched sample was cut into 12 equal pieces, which was further de-stained using 40% ACN (acetonitrile) and 40 mM NH_4_HCO_3_ (ammonium bicarbonate) at a ratio of 1:1 (*v*/*v*), respectively. The de-stained gel bands were reduced with 5 mM dithiothreitol (DTT) in 40 mM NH_4_HCO_3_, followed by alkylation with 20 mM iodoacetamide in 40 mM NH_4_HCO_3_. Overnight digestion of gel bands was carried out using 12.5 ng/μl trypsin (modified sequencing grade; Promega, USA) at 37°C. Further, peptide extraction was done from gel pieces, lyophilized, and desalted using zip tip (Millipore, Germany) following the manufacturer's instruction and stored at −80°C for peptide identification by LC-MS/MS.

### *In-solution* Tryptic Digestion

For in-solution digestion, 500 μg of pooled protein samples (50 μg each from 10 animals pooled from different stages of lactation) from TCA/acetone precipitated protein samples were processed separately. Further, 45 mM DTT (dithiothreitol) was dissolved in 50 mM NH_4_HCO_3_ (ammonium bicarbonate) to reduce disulfide bonds followed by alkylation of cysteine residues using 10 mM IAA (iodoacetamide) in 50 mM NH_4_HCO_3_ (ammonium bicarbonate). Digestion was carried out overnight using trypsin (1:100) at 37°C. The reaction was subsequently stopped with 10% TFA (trifluoroacetic acid), and peptides were further vacuum dried. Samples were fractionated into 24 fractions, further desalted by zip tip, and further subjected for peptide identification by LC-MS/MS (22).

### Electrospray Ionization Tandem Mass Spectrometry LC-MS/MS Analysis

The lyophilized peptide fractions were reconstituted in 0.1% formic acid in LC-MS-grade water and subjected to nano-LC (Nano-Advance; Bruker, Germany) followed by identification in captive ion source (Bruker Captive Spray tip) spray-in Maxis-HD qTOF (Bruker) mass spectrometer (MS) with high mass accuracy and sensitivity. The peptides were enriched in nano-trap column (Acclaim Pep Map, particle size 5 μm, pore size 100 Å; Thermo Scientific) and eluted on to nano-analytical column (Kaya Tech HIQ SIL C_18_HS/3, 0.1 × 150 mm, 3 μm particle size, and 200 Å pore size). The peptide elution was carried out using a linear gradient of 5–45% acetonitrile at 400 nl/min flow rate in a total run time of 135 keeping the solvent system as follows: solvent A, 100% water in 0.1% formic acid; and solvent B, 100% acetonitrile in 0.1% formic acid. Positive ions were generated by electrospray, and the q-TOF was operated in data-dependent acquisition mode to automatically switch between MS and MS/MS acquisition. Precursor ion TOF MS survey scan was acquired with a range of 300–1,800 *m*/*z* with resolution R = 75,000. Q1 sequentially selects six most intense precursor ions for fragmentation using collision-induced dissociation for MS-MS analysis with a fixed cycle time of 3 s along with 2 min of release for exclusion filter (Data acquisition otof software, version 24.8; Bruker Daltonics).

### Data Processing and Analysis

The vendor-created.d file format was analyzed with three different search engines that are Mascot, Comet, and Tandem for high confidence identification of the profiled whey proteome. For Mascot (Matrix Science, UK, version 2.4.1), we used the ProteinScape platform (version 2.0). Initially, the peak lists were generated through Hyster (Bruker Daltonics) to make spectral data in the form of mgf format (Mascot Generic Files). Resultant mgf files were used for the identification of proteins using the UniProt database downloaded April 3, 2019 along with usual contaminant proteins for spectra examination. The parameter for MS/MS ion search contains tryptic digested sites with two missed cleavage allowed, flexible modification on amino acids—methionine for oxidation, N-terminal acetylation, Gln-pyro Glu, and Glu-pyro Glu, while static modification of cysteine as carboxy amidomethylation or propionamide. The peptide precursor ion tolerance was 20 ppm, with MS/MS tolerance of 0.01 Da. The “ion score cutoff” was set to 30, thereby eliminating the lowest quality matches and minimum peptide length as six amino acid residues. To increase the confidence and remove the false-positive identification, a 1% false discovery rate (FDR) was used at equal peptide and protein levels. The decoy reversed sequences database was included for the calculation of the FDR.

The data were also re-searched in Trans-Proteomic Pipeline (version 5.1.0) for Comet and Tandem search engines. The in-depth procedure for performing the analysis was reported in Suhail et al. (23). Briefly, in the first step, the raw data were transformed to open format mzML files. The converted files were searched using the TPP pipeline keeping the aforementioned common database and the search parameters. However, on a different note, TPP initially performs the peptide assignments using Tandem and Comet search engine. In addition, PeptideProphet and ProteinProphet algorithms were employed in the pipeline to calculate the probability values for both independently examined peptides and the corresponding proteins. The exact mass model for high probability in PeptideProphet was utilized to boost the confidence for peptide associations and also to promote the definite possibility of peptides. Extra protein validation level was performed applying both PeptideProphet and ProteinProphet scores, where the protein was confirmed if it includes at least two top-ranked peptides with each peptide probability score above 95% (Supplementary Table 1). iProphet algorithm validated and merged all search engine results. This method takes as the input of PeptideProphet spectrum-level results from multiple LC-MS/MS runs and then computes a unique probability at the level of a unique peptide sequence (or protein sequence). This structure provides for the incorporation of outcomes from various search engines. It brings into account additional promoting determinants, including the number of sibling experiments distinguishing the equivalent peptide ions, the number of replicate ion identifications, sibling ions, and sibling modification states. A model of iProphet performance with regard to the abundance of correct entries vs. error is provided in Supplementary Table 1. An iProphet probability of higher than 0.9999 was accepted as the cutoff for the conclusive identification of the protein. For protein quantitation, ≥2 unique peptides per protein were estimated to ensure high-quality quantitation.

### Bioinformatics Analysis

All the graphical analyses were performed in the R environment using respective tools. Histograms, density scatter plots, and principal component analysis (PCA) were generated using the ggPlot2 package. All the identified proteins were mapped with UniProt bovine chromosomal proteome information and parsed to create files appropriately formatted for input to Circos. The Gene Ontology (GO) categories were analyzed using the DAVID bioinformatics resources, and only the genes with a *p* adjusted value (FDR) of <0.05 were included and subsequent GO term plotted.

## Results

### Assessment of Different Methods for the Quality of Aqueous Milk Whey Protein Extraction

The present study aims to determine the comprehensive profile of bovine milk whey proteome in Indian zebu (*Sahiwal*) cattle. The method optimization using three different procedures (ultracentrifugation-acetone precipitation, CaCl_2_ precipitation at different concentrations, and TCA/acetone precipitation methods) for the isolation of the aqueous whey fraction showed that TCA/acetone precipitation performed best and resulted in the identification of maximum peptides/proteins in quality check LC-MS/MS runs (data not shown). The SDS-PAGE profiles for different protein extraction procedures and method optimization for milk whey samples also showed a better profile of the TCA/acetone precipitation method in comparison with others (Supplementary Figures 1A,B). Before running the whole experiment, we performed the quality assurance test; we ran the 2D gel for the TCA/acetone precipitated whey and identified the clear sorted pattern of spots (Supplementary Figure 2), suggesting its suitability for further in-depth proteome analysis.

### Protein Identification by LC-MS/MS

**Figure 1:**
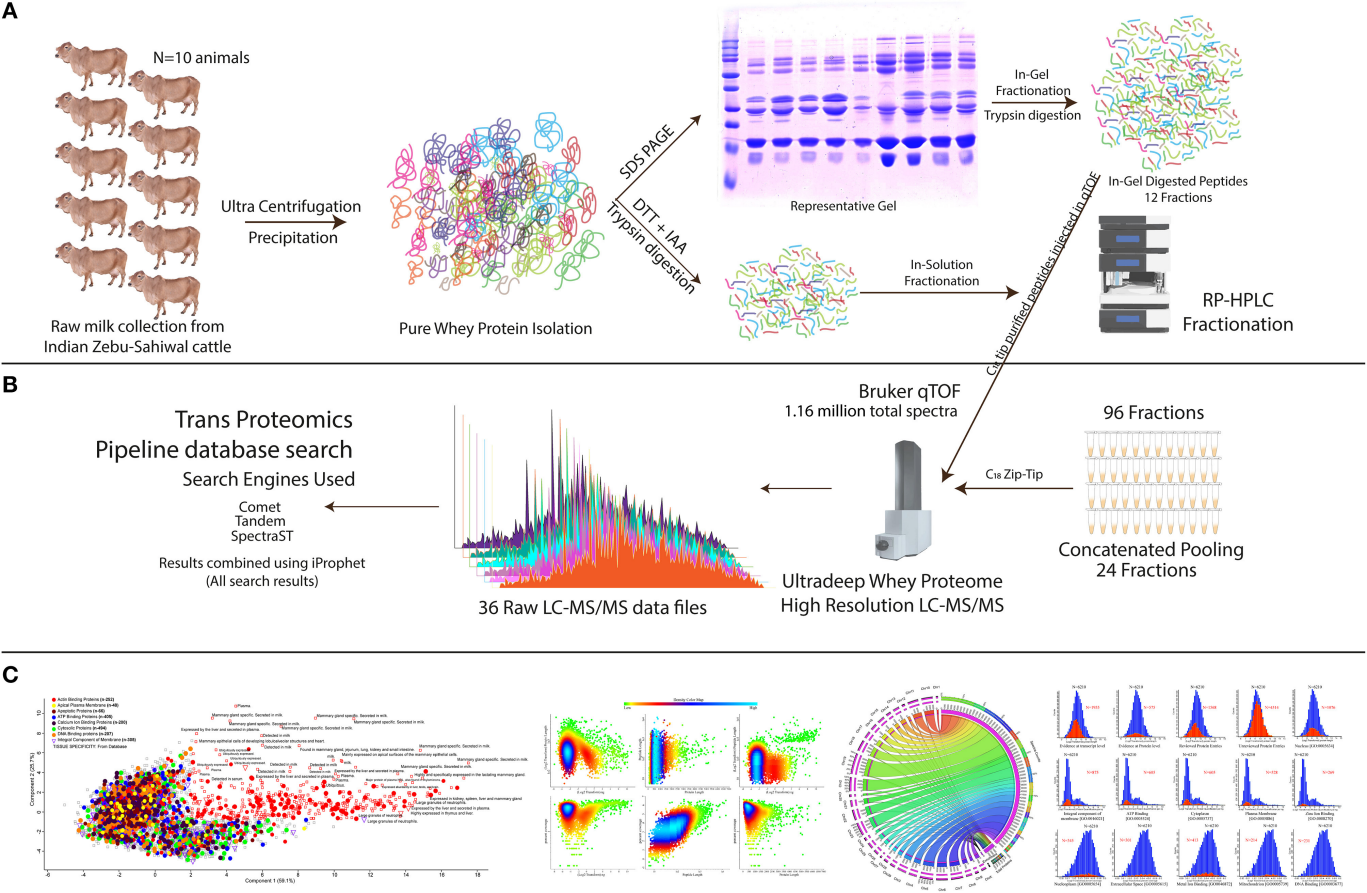
Experimental setup and systemic proteomics workflow for the comprehensive discovery of bovine milk whey proteome. **(A)** From 10 individual animals and pooled milk samples, the milk whey proteome is isolated and purified using ultracentrifugation and TCA/acetone precipitation strategy. In-gel fractionation through SDS-PAGE and in-sol digested peptides were subjected to C18 chromatography for long gradient 96 fractionation (see Materials and Methods). **(B)** C18 separated fractions were concatenated, mixed, and analyzed using a high-resolution qTOF instrument. All the raw data were extracted, and the resulting peptides and proteins were analyzed using the TPP pipeline utilizing Comet, Tandem, and SpectraST search engines, and the obtained results were compiled using iProphet algorithm as described in Materials and Methods section. **(C)** Brief description of the data analysis; detailed information is provided in the Materials and Methods section. For determination of the high confidence bovine whey proteome, data were filtered using the stringent parameter. Evidence of the proteome at the different levels was determined along with the identification of the different Gene Ontology (GO) classes in it.

We employed the optimized bovine milk whey extraction workflow for the identification of ultra-deep proteome (Supplementary Figures 1, 2). We ran the SDS-PAGE whey proteome profile of 18 animals separately (Supplementary Figure 3) and selected 10 animals whose profile was matching almost similar for sample pooling. In this way, we can determine the whey proteome profile of the population and neglected the animal-to-animal variations. The isolated proteome of indigenous zebu (*Sahiwal*) milk whey samples were fractionated using a combined approach: (1) *in-gel digestion* LC-MS/MS methods and (2) *in-solution digestion* LC-MS/MS methods. Samples prepared from *in-gel* and *in-solution* tryptic digestion were subjected to high-resolution qTOF, nano-LC-MS/MS (Figure 1). An outstanding feature of analyzing this dataset is the conversion of open file format and its interchange data processing between modules (i.e., comet, tandem, SpectraST) under the umbrella of Trans Proteomics Pipeline (TPP). Finally, the total combined run of 36 fractions resulted in the identification of 1,154,123 spectra, 57,286 peptides, and 6210 proteins. The authenticity and confidence of the identified proteome were also confirmed through cross-mapping the proteome information using three independent proteomics software (TPP and Protein Scape) (Supplementary Tables 1, 2). Only the 6,210 common proteins identified in all the three platforms were selected for further analysis. All the proteins were selected at <1% FDR and having the protein prophet and iProphet value of 0.9999 and 0.95, respectively. Next, we chose the MS2-based Normalized Spectral Index for the quantification of individual proteins using the StPeter algorithm implemented in TPP for whey proteome quantitation. It allows the identification of individual proteins in the sample.

**Figure 2:**
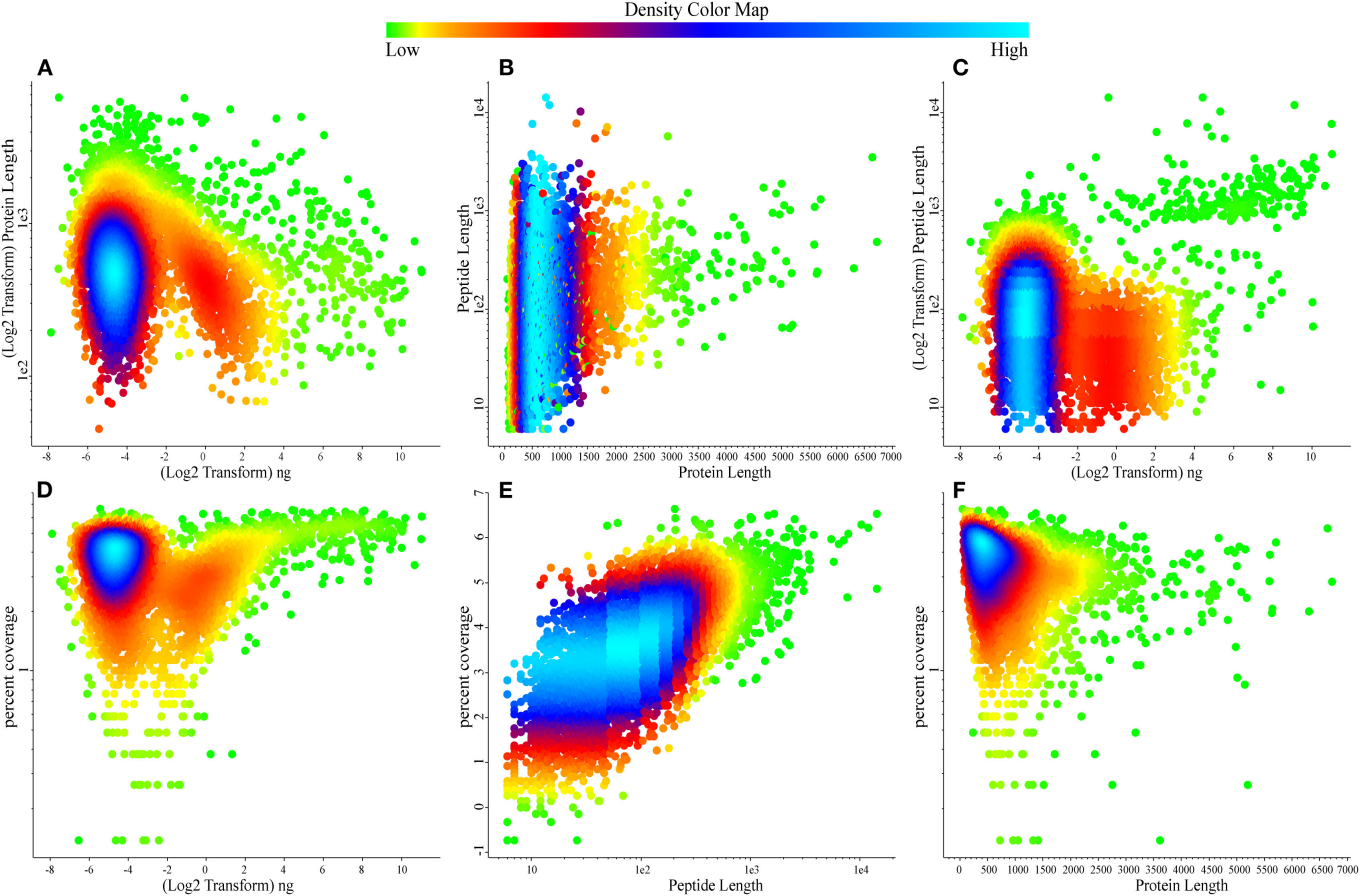
Scatter relationship plots describing the density of attributes over the bovine whey proteome data. **(A)** Protein lengths over quantification values. **(B)** Peptide length over protein length. **(C)** Peptide length over quantification values. **(D)** Protein percentage coverage over quantification values. **(E)** Protein percentage coverage over peptide length. **(F)** Protein percentage coverage over protein length. Color map reports the density of the values in the data, green reports the low, dense data points while sky blue reports highly dense data points.

To date, this is the largest comprehensive whey proteome dataset. Our next aim was to determine the quality pattern of the proteins present in the whey. We plotted density plots to identify the information; most of the densely populated proteins were determined in the concentration range of 0.0156–0.0625 ng (Figures 2A,B) with the identified high-density percentage coverage of 20–65% (Figures 2C,D) in whey proteome. The protein length is ranging in between 500 and 800 amino acid residues (Figure 2F) with the identification of tryptic peptides ranging between 7 and 15 amino acid residues (Figure 2E).

### Chromosomal Mapping of Bovine Whey Proteome

**Figure 3:**
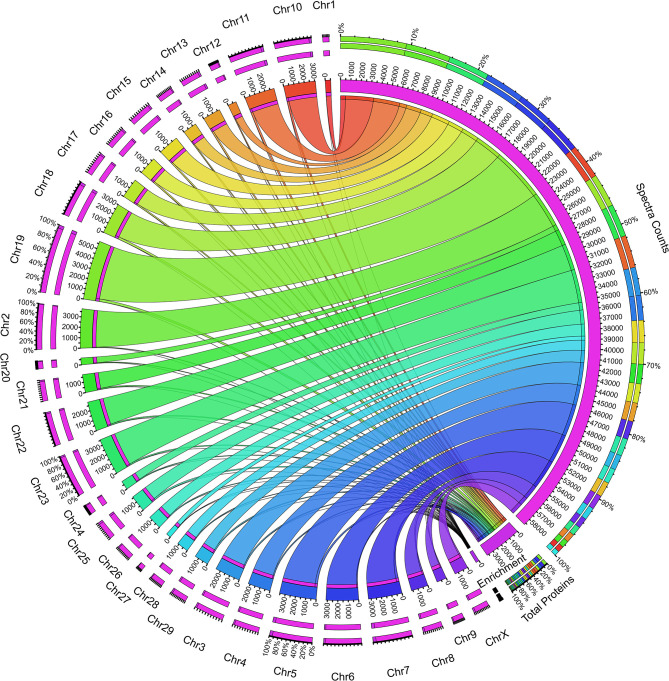
Circos plot showing the whey protein expression mapped to individual chromosomes in the bovine genome. The right inner pink ring indicates the total spectra collected during the proteome analysis. The connecting set of ribbons describes the collection of spectra identified from individual chromosomes, and its width showed the number of spectra assigned. The higher the number spectra, the broader the ribbon width and vice versa. The bottom of the plot describes the total proteins assigned and the enrichment value based on the protein coded for the respective chromosome. The left outer ring describes the chromosomes and the number of protein assignment, which expresses bovine whey.

Next, we sought to determine the expression of the proteins from the individual chromosomes of the genome. We performed the chromosomal mapping analysis creating a Circos plot based on the UniProt data. Total spectra and protein information fetched from the search engine for identification of the proteins were mapped using the UniProt database. We found the contribution of all the 29 + X chromosomes in the whey proteome, but the analysis resulted in interesting facts such as the uneven distribution of the protein expression in the chromosome-wise manner. Chromosome 1 showed a minimum expression of 14 proteins, whereas chromosome 19 expressed 250 maximum proteins (Figure 3); the average expression number of proteins per chromosomes is 123. To make the analysis unbiased, we calculated the enrichment percentage of all the chromosomes which is the division of the total proteins assigned in the database to the actual number of proteins identified in the mapping analysis; however, the results are the same (Supplementary Table 3). The factors responsible for such high expression of chromosome 19 and very low expression of chromosome 1 contributing to the whey proteome are unknown. Nonetheless, further studies on genome-wide proteome analysis of mammary gland cells are required to answer these questions.

### Functional Significance of Bovine Milk Whey Proteins

To understand the overall function of bovine whey protein/proteome, we characterize the subcellular localization using the program ngLOC utilizing the n-gram-based Naïve Bayesian classification model for the fixed-length peptide sequences density distributions over each distinct subcellular location (Supplementary Table 4). The results showed the identification of 11 different organelle classification while maximum proteins 46.13% (3,012 proteins) in whey are annotated to come from nucleus followed by cytoplasm 18.63% (1,220 proteins) and plasma membrane 17.20% (1,123 proteins). Further, we performed the ontological gene classification and found the majority of proteins in functional GO terms were nuclear proteins (46.13%) followed by proteins present in the cytoplasm (18.68%), placenta (17.2%), exosomes (7.08%), and Golgi bodies (1.18%). The cellular component GO enriched term reveals the identification of 18.92% proteins in the cytoplasm, 17.41% in the nucleus, 16.5% proteins were phosphoproteins, and 15.5% were present in extracellular exosomes. The majority of proteins had a functional role in metal ion binding (9.88%) and ATP binding (9.5%) (Supplementary Table 5).

Molecular functions in terms of protein percentage reveal the involvement of the majority of proteins in ATP binding (9.5%) followed by metal ion binding, RNA binding, zinc ion binding, DNA binding, and transcription factor activity. The proteins in biological process class were majorly identified in transcription, DNA template, oxidation–reduction process, intracellular signal transduction, and regulation of transcription. In similar terms, the majority of proteins in the KEGG pathway were present in metabolic pathways, PI3K, Akt, MAPK, and RAS signaling pathways. Also, several proteins have a role in pathways related to cancer, focal adhesion, regulation of actin cytoskeleton, and endocytosis. In the SMART term, the majority of proteins are SMW382: AAA. In Interpro term counts, the majority of protein percentage was found in P-loop containing NTP hydrolase, Protein kinases like domain, Homeodomain, and Serine/threonine–protein kinase active site. Clustering the proteins in terms of tissue specificity, milk proteins revealed expression in the mammary gland, milk, fetal skin and muscle, brain, and oviduct.

In Reactome term counts, 1.09% proteins were involved in platelet degranulation, followed by ubiquinilation and proteasome degradation, anchoring of the basal body to the plasma membrane, cell adhesion, *in utero* embryonic development, oxidation–reduction process, and positive regulation of gene expression (Supplementary Figure 4).

**Figure 4:**
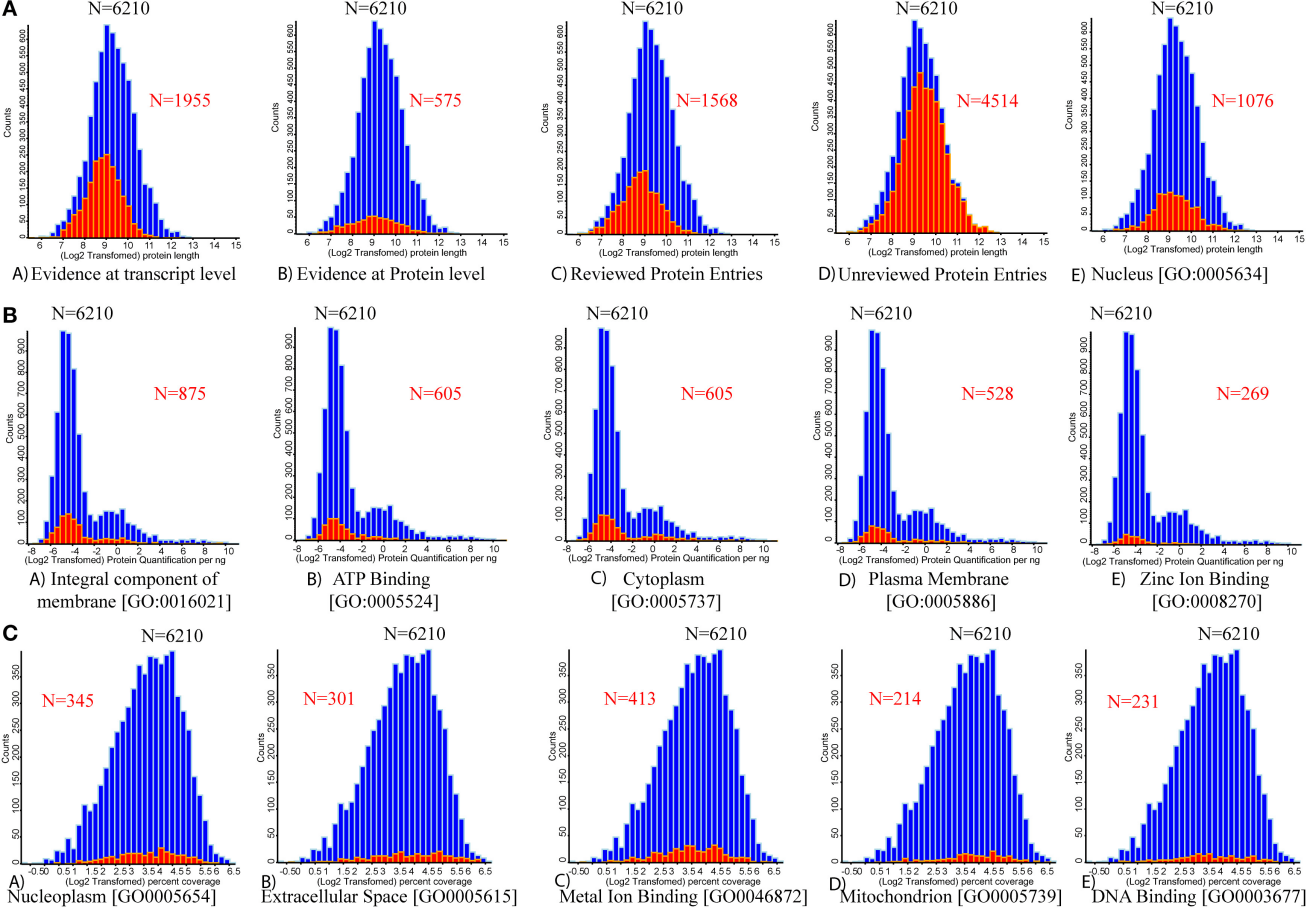
Histograms for the distributions of the protein lengths, protein quantification, and percentage coverage are presented. The vertical axis of the histogram presents the number of each categorical protein in each bin. **(A)** Distribution of varying protein length (full length) counts identified through database mapping to the number of proteins. (A) Evidenced at transcript level. (B) Evidenced at protein level. (C) Reviewed UniProt database protein entries. (D) Un-reviewed UniProt database protein entries. (E) Protein associated with nucleus origin. **(B)** Mapping the number of protein quantification values for five high abundant UniProt-based GO terms. (A) Integral component of membrane. (B) ATP binding. (C) Cytoplasm. (D) Plasma membrane. (E) Zinc ion binding proteins. **(C)** Mapping the number of individual protein percentage coverage for the next five high abundant UniProt-based GO terms. (A) Nucleoplasm. (B) Extracellular space. (C) Metal ion binding. (D) Mitochondrion. (E) DNA binding. Red color overlapped histogram presents the respective mapped attribute over the whole bovine whey proteome data (*n* = 6,210).

Bioactive proteins present in the whey fraction are involved in a wide range of physiological activities, including anti-inflammatory effects and protection against pathogen-induced intestinal inflammation (24). The relationship between counts vs. log_2_-transformed protein length reveals that out of 6,210 proteins, 1,955 proteins had evidence at transcript levels; however, 575 proteins have evidence at the protein level. Out of 6,210 entries, 1,568 protein entries are reviewed, and 4,514 entries are unreviewed (Figure 4). The relationship between counts vs. log_2_ protein quantification per ng reveals 875 proteins as integral components of the membrane (GO: 0016021). The GO terms reveal that 1076 proteins (GO: 0005634) were present in the nucleus, 605 in the cytoplasm (GO: 0005737), 528 in the plasma membrane (GO: 0005886), 345 proteins in nucleoplasm (GO: 0005654), 301 in extracellular space (GO: 0005615), and 214 in mitochondria (GO: 0005739). Some other proteins involved in different metabolic processes include 605 proteins in ATP binding (GO: 0005524), 269 proteins in zinc ion binding (GO: 0008270), 413 proteins in metal ion binding (GO: 0046872), and 231 proteins in DNA binding (GO: 0003677).

### PCA Score Plots for Milk Whey Proteome

**Figure 5:**
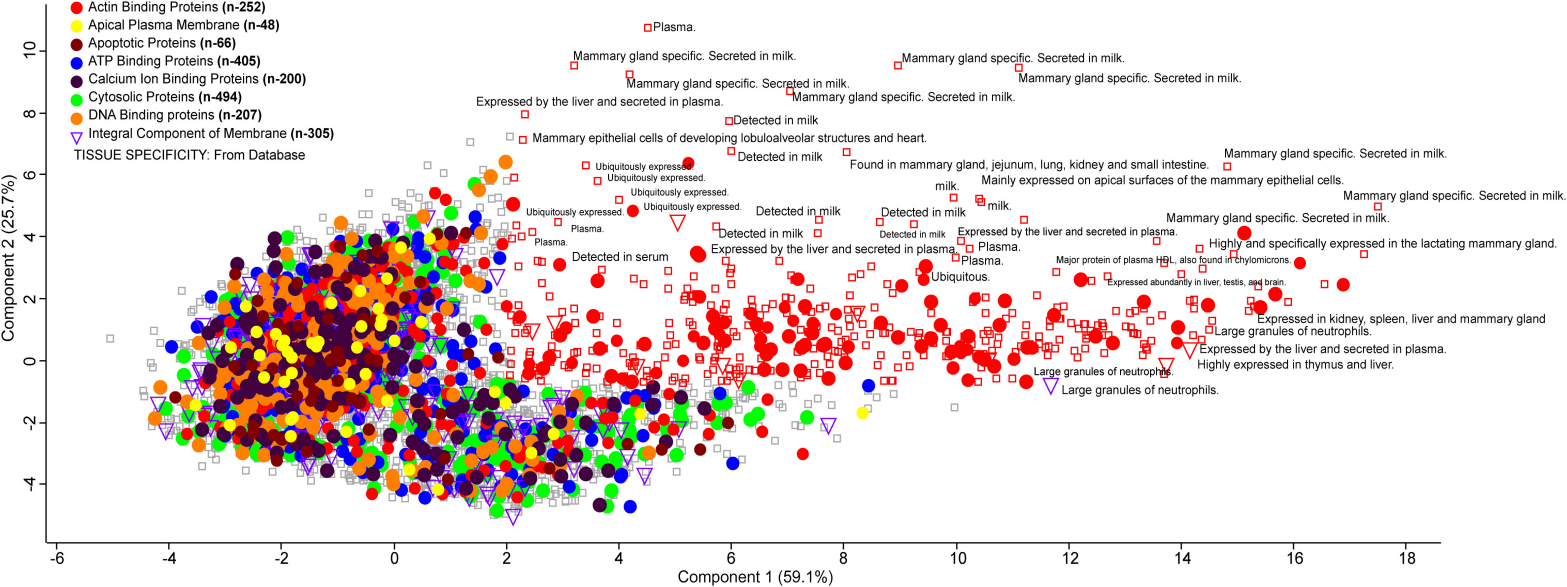
PCA plot describing the orthogonal dispersion of the proteins reported in the deep whey proteome analysis. Color coding indicates the different class of major proteins identified in the whey proteome data. All the proteins identified to specific tissue from the UniProt database are reported with a rectangle.

Milk whey proteins can be graphically represented using a PCA score plot where two components explain maximum variability. It is used as a data reduction tool where the score plot represents a dataset of 6,210 proteins. These were assigned as actin-binding proteins (*n* = 252), apical plasma membrane (*n* = 48), apoptotic protein (*n* = 66), ATP binding protein (*n* = 405), Ca ion binding protein (*n* = 200), cytosolic protein (*n* = 494), DNA binding protein (*n* = 207), and integral component of membrane (*n* = 305). The score plot classifies the whey proteins based on their involvement in several metabolic, biochemical, and cellular processes. Actin-binding proteins are maximally distributed, and the majority of proteins were mammary gland specific, secreted in milk. Some of the integral components are found in the large granules of neutrophils with their possible role in immunoregulation (Figure 5).

Many of the milk bioactive proteins and peptides are also known to exhibit multifunctional physiological properties. In the present study, the majority of proteins with a high score as beta-lactoglobulin, lactoperoxidase, caseins, alpha-lactalbumin, alpha-2 macroglobulin, complement C3, lactotransferrin, alpha-S2 and S1 casein, xanthine oxidase, serotransferrin, and lactadherin were found to play a role in immune regulation and host defense. The top 60 lowest abundant proteins identified with high confidence levels and significant scores are shown in Table 1. The majority of the high score proteins have a role in immune status and host defense against invading microbes. However, the high abundant proteins like complement C3, lactoferrin, lactoperoxidase, and xanthine oxidase proteins exhibit immunomodulatory and anti-inflammatory properties. Polymeric immunoglobulin receptor (PIGR) and osteopontin proteins play a role in the innate immune system (25). Heat shock proteins (HSP 70) were found to be involved in pathways related to thermotolerance and help in maintaining homeostasis against adverse climatic conditions in livestock (26).

**Table 1:** Top 60 low abundant proteins identified in whey proteome with respective database information, molecular function, cellular component, biological process, Prosite, Pfam, InterPro, SMART, and SUPFAM.

**Protein**	**Protein names**	**Gene symbol**	**ng**	**Molecular function**	**Cellular component**	**Biological process**	**Prosite**	**Pfam**	**InterPro**	**SMART**	**SUPFAM**
F1MCK2	AHNAK nucleoprotein	AHNAK	0.074		Costamere	Regulation of RNA splicing	PS50106		IPR001478	SM00228	SSF50156
E1BPD7	Marker of proliferation Ki-67	MKI67	0.066				PS50006	PF00498	IPR000253	SM00240	SSF49879
F1N415	Piccolo presynaptic cytomatrix protein	PCLO	0.075	Metal ion binding	Presynaptic active zone	Synapse assembly	PS50004	PF00168	IPR000008	SM00239	SSF50156
E1BGB0	Kinesin family member 13B	KIF13B	0.054	14-3-3 protein binding	Axon	Microtubule-based movement	PS00845	PF01302	IPR036859	SM01052	SSF49879
F1MV51	APC regulator of WNT signaling pathway	APC	0.094	Beta-catenin binding	Beta-catenin complex	Cell cycle arrest	PS50176	PF05972	IPR026836	SM00185	SSF48371
E1BLA0	Shugoshin 2	SGOL2	0.057		Centromeric region	Meiotic sister chromatid cohesion			IPR026706		
E1BKT9	Desmoplakin	DSP	0.018	Cell adhesion molecule binding	Plasma membrane	Adherens junction organization	PS50002	PF00681	IPR028462	SM00250	SSF75399
E1BPB1	Kinesin family member 24	KIF24	0.093	ATPase activity	kinesin complex	Microtubule-based movement	PS00411	PF00225	IPR027640	SM00129	SSF47769
L8IEV2	Dynein heavy chain 1, axonemal	M91_21125	0.078	ATPase activity	Dynein complex	Cilium movement		PF12774	IPR035699		SSF52540
E1BHP0	Alpha-mannosidase	MAN2B2	0.095	Alpha-mannosidase activity	Vacuolar membrane	Mannose metabolic process		PF09261	IPR011013	SM00872	SSF74650
L8IV13	Leucine-rich repeat-containing protein KIAA1731	M91_18003	0.083	Microtubule binding	Centriole	Positive regulation of centriole elongation		PF15309	IPR029299		
L8HSL9	WD repeat-containing protein 87	M91_17430	0.085				PS50082	PF00400	IPR015943	SM00320	SSF50978
E1BKZ5	Golgin B1	GOLGB1	0.084		cis-Golgi network	Protein localization to pericentriolar material			IPR026202		
F1N2K7	Phosphoinositide kinase, FYVE-type zinc finger containing	PIKFYVE	0.033	1-Phosphatidylinositol-3-phosphate 5-kinase activity	Cell–cell junction	Intracellular signal transduction	PS50186	PF00118	IPR002423	SM00049	
E1BC24	Midasin	MDN1	0.062	ATPase activity	Cytosol	Ribosomal large subunit assembly	PS50234	PF07728	IPR003593	SM00382	SSF52540
E1BKC4	Nipped-B protein	NIPBL	0.071	Chromatin binding	Chromatin	Brain development		PF12765	IPR011989		SSF48371
L8IT59	Vacuolar protein sorting-associated protein 13A	M91_05527	0.081					PF09333	IPR015412		
E1BKI3	Nitric oxide synthase (EC 1.14.13.39)	NOS1	0.065	Calmodulin binding	Cytosol	Arginine catabolic process	PS51384	PF00667	IPR003097	SM00228	SSF50156
E1B9N6	Envoplakin	EVPL	0.035	Intermediate filament binding	Cornified envelope	Epidermis development		PF00681	IPR041615	SM00250	SSF75399
E1BPX1	Vacuolar protein sorting 13 homolog C	VPS13C	0.103		Cytosol	Mitochondrion organization		PF09333	IPR015412		
O46382	Brefeldin A-inhibited guanine nucleotide-exchange protein 1	ARFGEF1	0.091	ARF guanyl-nucleotide exchange factor activity	Cytosol	Endomembrane system organization	PS50190	PF16213	IPR016024	SM00222	SSF48371
F1MC51	ATP binding cassette subfamily B member 5	ABCB5	0.067	ATPase activity	Plasma membrane		PS50929	PF00664	IPR003593	SM00382	SSF52540
E1BHT5	Ubiquitin protein ligase E3 component n-recognin 4	UBR4	0.065	Ubiquitin-protein transferase activity	Centrosome	Ubiquitin-dependent protein catabolic process	PS51157	PF13764	IPR016024	SM00396	SSF48371
E1BCI2	E3 ubiquitin protein ligase (EC 2.3.2.27)	RNF40	0.061	Metal ion binding	HULC complex	Histone H2B ubiquitination	PS00518		IPR013956	SM00184	
E1BL95	Centromere protein J	CENPJ	0.037	Identical protein binding	Centriole	Microtubule polymerization		PF07202	IPR033068		
E1B7W1	Thyroid hormone receptor associated protein 3	THRAP3	0.092			RNA splicing		PF15440	IPR026667		
F1N137	Inositol 1,4,5-trisphosphate receptor type 2	ITPR2	0.094	Inositol 1,4,5 trisphosphate binding	Endoplasmic reticulum		PS50919	PF08709	IPR014821	SM00472	SSF100909
F6RF21	Structural maintenance of chromosomes flexible hinge domain containing 1	SMCHD1	0.047	ATPase activity	Barr body	Double-strand break repair		PF06470	IPR036890	SM00968	SSF55874
F1MHT1	Amylo-alpha-1, 6-glucosidase, 4-alpha-glucanotransferase	AGL	0.047	4-Alpha-glucanotransferase activity	Cytoplasm	Glycogen biosynthetic process		PF06202	IPR008928		SSF48208
L8J1D8	Eukaryotic translation initiation factor 3 subunit B (eIF3b)	EIF3B	0.072	Translation initiation factor activity	43S complex	Cytoplasmic translation initiation complex	PS50102	PF08662	IPR011400	SM00360	SSF54928
F1MJ95	Unc-80 homolog, NALCN channel complex subunit	UNC80	0.081	Cation channel activity	Axon	Cation homeostasis		PF15778	IPR031542		
E1B9N8	Lysine methyltransferase 2D	KMT2D	0.033	Histone binding	MLL3/4 complex	RNA polymerase II	PS51805	PF05965	IPR034732	SM00542	SSF47095
F1MWK8	Protein tyrosine kinase 7 (inactive)	PTK7	0.089	ATP binding	Cell–cell junction	Actin cytoskeleton reorganization	PS50835	PF07679	IPR007110	SM00409	SSF48726
E1BCV4	Nucleoporin 98	NUP98	0.059	mRNA binding	Kinetochore	Nuclear pore complex assembly	PS51434	PF04096	IPR037665		SSF82215
A4IFK4	Synaptopodin 2	SYNPO2	0.076	Actin binding	Cytoskeleton	Actin bundle assembly	PS50106	PF00595	IPR001478	SM00228	SSF50156
A6QLI5	Melanoma-associated antigen D4 (MAGE-D4 antigen)	MAGED4	0.094				PS50838	PF01454	IPR037445	SM01373	
E1BLB6	Protein kinase, DNA-activated, catalytic subunit	PRKDC	0.086	ATP binding	DNA-dependent kinase- ligase 4 complex	Activation of innate immune response	PS51189	PF02259	IPR016024	SM01343	SSF48371
F1MGR3	Arginine-glutamic acid dipeptide repeats	RERE	0.100	Chromatin binding	Histone deacetylase	Branching of a nerve	PS51038	PF03154	IPR002951	SM00439	SSF46689
A6H737	Lysyl oxidase homolog 2 (Lysyl oxidase-like protein 2)	LOXL2	0.080	Calcium ion binding	Basement membrane	Cellular protein modification process	PS00926	PF01186	IPR001695	SM00202	SSF56487
A6QQJ9	GPRASP1 protein	GPRASP1	0.085					PF04826	IPR006911		SSF48371
F1MZM7	Synapse defective Rho GTPase homolog 2	SYDE2	0.054			Signal transduction	PS50238	PF00620	IPR035892	SM00324	SSF48350
A0JN33	Kin of IRRE like 3	KIRREL3	0.083		Plasma membrane		PS50835	PF07679	IPR007110	SM00409	SSF48726
F1MNU5	Phospholipase A2	PLA2G4F	0.078	Calcium-dependent phospholipase A2	Cytoplasm	Arachidonic acid secretion	PS50004	PF00168	IPR016035	SM00239	SSF52151
E1B949	FAT atypical cadherin 4	FAT4	0.086	Calcium ion binding	Apical part of cell	Branching involved in ureteric bud	PS00010	PF00028	IPR002126	SM00112	
F1MZU6	Collagen alpha-3(IV) chain	COL4A3	0.048	Extracellular matrix	Collagen	Apoptotic process	PS51403	PF01413	IPR008160	SM00111	SSF56436
E1BEB3	Histone acetyltransferase	KAT6A	0.102	DNA binding	Cytosol	Cellular senescence	PS51504	PF01853	IPR016181	SM00526	SSF46785
E1BKZ9	Sortilin	SORT1	0.070		Cytosol	Endocytosis		PF15902	IPR031777	SM00602	
L8HZQ1	Kielin/chordin-like protein	M91_00882	0.063				PS01208	PF00093	IPR036084	SM00832	SSF57567
F1MG90	WDFY family member 4	WDFY4	0.023				PS50197	PF02138	IPR016024	SM01026	
L8J3H5	Sperm-associated antigen 1	M91_19629	0.096				PS50005	PF13877	IPR025986	SM00028	SSF48452
E1BHN4	RING-type domain	RNF213	0.063	ATPase activity	Cytoplasm	Wnt signaling pathway	PS00518	PF00097	IPR003593	SM00382	SSF52540
F1MFU7	Zinc finger protein 862	ZNF862	0.069	Nucleic acid binding		Transcription	PS50805	PF05699	IPR008906	SM00349	SSF10964
E1BNA3	Adaptor related protein complex 4 subunit epsilon 1	AP4E1	0.064		AP-4 adaptor complex	Vesicle-mediated transport		PF01602	IPR017109	SM01356	SSF48371
P98167	SCO-spondin	SSPO	0.044	Peptidase inhibitor	Extracellular	Cell adhesion	PS01225	PF08742	IPR006207	SM00832	KOG1215
E1BEJ9	NLR family apoptosis inhibitor	NAIP	0.056	ATP binding	Plasma membrane	Apoptotic process	PS50143	PF00653	IPR003593	SM00382	SSF52540
F1MFH3	FRAS1 related extracellular matrix protein 2	FREM2	0.055		Basement membrane	Cell communication	PS51854	PF03160	IPR038081	SM00237	SSF141072
E1B754	Microtubule associated serine/threonine kinase 2	MAST2	0.086	ATP binding	Microtubule cytoskeleton	Cytoskeleton organization	PS51285	PF08926	IPR000961	SM00228	
E1BP05	Usherin	USH2A	0.092	Collagen binding	Apical plasma membrane	Animal organ morphogenesis	PS01248	PF00041	IPR013320	SM00180	SSF49265
A3KN18	CEBPZ protein	CEBPZ	0.067			Ribosome biogenesis		PF03914	IPR016024		SSF48371

## Discussion

To date, to the best of our knowledge, the current report is the most comprehensive characterization of minor bovine milk proteome. Bovine milk is a complex body fluid containing various secreted proteins and is an important source of human nutrition. However, the minor immune-related host defense proteins have not been wholly characterized and need to be explored exhaustively. Milk-derived bioactive peptides present in whey and casein have potential roles as health-promoting agents against cancer, diabetes, hypertension, osteoporosis, and several other disorders (27). Whey proteins can serve promising health benefits in terms of their ability to act as antioxidant, antihypertensive, anti-tumor, anti-viral, anti-bacterial, and hypo-lipidemic (28). Bioactive peptides present in milk exosomes, colostrum, milk fat globular membrane, skimmed milk, and whey provide information on generating large bovine milk proteome datasets.

The present study was designed with the aim to explore the in-depth milk whey proteome of Indian zebu (*Sahiwal*) cattle, which was not studied earlier. Indian zebu (*Sahiwal*) cattle have been acclaimed for the potential to disease resistance and adaptability for heat stress as compared with exotic *Bos taurus* cattle. For proteomics analysis purpose, different protocols for milk protein extraction were attempted including acetone precipitation of full cream milk (20), ultracentrifugation to pellet caseins (7), ammonium sulfate precipitation of caseins to isolate serum (29), and acetic acid removal of caseins to isolate whey proteins (30). Further, optimization of protein extraction methods in skimmed milk of Holstein and Jersey breeds was studied (21), where they compared three protein extraction procedures that were urea-based buffer, TCA/acetone, and methanol/chloroform. The results reported the methanol/chloroform method as the best-optimized method for peptide identification in cattle breeds. In our study, we optimized three methods for protein extraction, which includes ultracentrifugation followed by acetone precipitation, CaCl_2_ precipitation at different concentrations, and TCA/acetone precipitation method. Out of these three methods, TCA/acetone precipitation was found to be the best-optimized method with maximum peptide identification in comparison with other protein extraction methods.

In an attempt to generate the proteome map, Smolenski et al. used bovine skimmed milk, whey, and milk fat globular membrane during peak lactation, colostrum, and mastitis stages. The results identified only 53 minor milk host defense proteins with 2,903 peptides (2). The latest report on differentially expressed changes in lactation and parity stage-specific whey proteome determined 103 proteins on primiparous cows (31). According to earlier reports, milk proteins from exosomes, milk fat globular membrane, skimmed milk, and whey have a nutraceutical role (32). Putative biomarkers of negative energy balance from milk proteins of early lactation in dairy cows were identified (33). The study found six milk proteins out of 59 proteins linked to metabolism and mammary gland cell proliferation. Another study was conducted on the characterization of bovine milk whey proteome during early lactation in Holstein and Jersey breeds by Tacoma et al. (18). They identified 935 low abundant proteins in skimmed milk fractions of the two breeds and highlighted the breed difference between proteome of Jersey and Holstein Friesian cows. However, no reports are available to date on milk whey profiling in Indian zebu cattle. Thus, the present study focuses on the global proteomic profiling of Indian zebu (*Sahiwal*) cattle, an important milch breed of the Indian subcontinent, to generate the proteomic profiling data for the first time. The findings suggest the identification and role of pathways related to activated immune response and stress tolerance homeostasis mechanisms. However, comparative proteomic analysis across different lactation stages was conducted in Malnad Gidda cattle (19) and Jersey and Kashmiri cattle (34). A recent report compiled an online database with 3,100 proteins from milk whey, milk fat globular membrane, and exosomes (35). Similarly, another separate study reported 4,654 cow milk proteins in five lactation stages to create the database. The stages were colostrum, early lactation, mid-lactation, peak lactation, and milk fractions consisting of exosomes, skimmed milk, and milk fat globular membrane (36).

Altogether, small sets of bovine milk proteome have been studied earlier, but none of them was able to provide the exhaustive proteome profile. The current report is the first time identification of 6,210 proteins in milk whey of Indian zebu (*Sahiwal*) cattle. It provides an in-depth identification of milk whey proteome to understand the role in human health and its biological significance. We determined proteome with the role in metal ion binding, zinc binding, and many more. Notably, from 6,210 proteins, a total of 1,568 proteins have UniProt reviewed protein entries, but the large majority (4,514) are unreviewed protein entries that have been validated by our data and could be further explored. Our bioinformatics analysis found that identified proteins are involved in various activities as ATP binding, DNA binding, metal ion binding, and zinc ion binding. Our fuzzy clustering analysis identified the presence of metal ion binding proteins, clusters in two independent databases Pfam and Panther, suggesting the ample presence of these proteins in whey (Supplementary Figure 5). The PCA revealed that actin-binding proteins are maximally distributed, and the majority of proteins were mammary gland specific, secreted in milk. Some of the integral components are found in the large granules of neutrophils with their possible role in immunoregulation.

The cellular component–centered gene ontology analysis reveals the involvement of major identified proteins in the nucleus, cytoplasm, plasma membrane, nucleoplasm, and mitochondria. Based on the interactive studies of whey proteins with metal ions, our whey proteome dataset proposed that it has potential applications in nutraceuticals and food industry (37). The metal ion binding properties of whey proteins could be highly useful and provide a way for designing functional foods. Purification of whey proteins such as lactoferrin which is known for its anti-bacterial and antimicrobial role can be utilized as a nutritional and dietary supplement as well as synergetic treatment in patients with anemia, kidney disease (38), and also in cancer (39). The low abundant minor milk proteins have a potential role in host defense and immune regulation. Several studies reviewed the role of immunoglobulins in milk for health and immunity (40–42). The presence of antimicrobial proteins and peptides such as beta-defensin, cathelicidins, complement proteins, and several other minor milk whey proteins were reported (42–46). In our study, we identified several minor proteins as cathelicidin 1 and 2, gelsolin, interferon 12 subunit alpha, fatty acid-binding protein, nucleobindin 2, transthyretin, claudin, haptoglobin, MFGE8 protein, polymeric immunoglobulin receptor, ubiquilin 4, and collectin 46 with different roles and biological significance (Table 1).

The complete proteome advocates that milk acts as an excellent medium for microbial growth that is evident from the enormous diversity of different high concentration peptides and proteins. It is the best source of nitrogen content for microbial growth. It allows the natural growth and habitat of beneficial microbes. Recently, we identified the occupancy of probiotics in fermented milk products and found a constant abundance (47). The determination of different attributes from the isolated microorganism showed positive results to classify them in the probiotics category (48). Combined genomics and proteomics analysis provide the landmarks for several genes and proteins coded in the genome for the probiotic attributes (49, 50). Hence, it strongly suggests that the whole milk supplementation allows the nourishment and abundant growth of healthy gut microbiota.

Apart from the significant milk proteins with a role in the innate immune system and host defense, numerous minor proteins were found to be overwhelming and found to be involved in response to regulation of immunity and inflammation. Quantification of the protein amount in ng reveals that significant whey proteins as alpha-lactalbumin, beta-lactoglobulin, beta-casein, ribonuclease pancreatic, albumin, and immunoglobulin chain proteins were identified in higher protein concentrations. Several other low abundant proteins (Table 1) as gelsolin, tetranectin, haptoglobin, cathepsin z, lysozyme, brain ribonuclease, peptidoglycan recognition protein 1, cathelicidin 2, vimentin, and others have been identified with a possible role in biological and physiological mechanisms.

## Conclusion

Milk proteome profiling was reported for the first time in Indian zebu (*Sahiwal*) cattle. Although studies were conducted in Holstein and Jersey (*B. taurus*) cattle, there is a lack of milk proteome profiling data in Indian zebu (*Sahiwal*) cattle. In this study, different protein extraction procedures as ultracentrifugation followed by acetone precipitation, CaCl_2_ precipitation at different concentrations, and TCA/acetone precipitation were optimized where TCA/acetone precipitation gave maximum peptide identification by LC-MS/MS. This study led to the identification of 6,210 proteins from *in-gel digestion* and *in-sol digestion*. All the generated proteomics raw files were analyzed with three different search engines, namely, Mascot, Comet, and Tandem, for high confidence identification of the profiled whey proteome. The majority of the proteins identified with a high score were found to play a role in immune regulation and host defense system implicating lesser disease susceptibility and better adaptability of Indian zebu (*Sahiwal*) cattle. The present study represents the first time reported *Sahiwal* milk proteome data in Indian zebu cattle, which could serve as a database for different cattle genetic resources of India.

## Data Availability Statement

The datasets presented in this study can be found in online repositories. The names of the repository/repositories and accession number(s) can be found in the article/Supplementary Material.

## Author Contributions

AC and SA equally designed, conducted data analysis, and wrote the article. AC, SA, SB, PR, and VV helped in designing and performing the experiments. SA performed the data analysis and created the illustrations for the article. SK and AM supervised the whole project and reviewed the article. All authors contributed to the article and approved the submitted version.

## Conflict of Interest

The authors declare that the research was conducted in the absence of any commercial or financial relationships that could be construed as a potential conflict of interest.

## Supplementary Material

Supplementary Figure 1 **(A)** Comparison of SDS-PAGE patterns of milk whey samples for optimization of different extraction procedures. 1D image of milk whey protein extraction procedures followed for optimization. (a) Ultracentrifugation followed by acetone precipitation. (b) CaCl_2_ precipitation method at different concentrations: 60, 90, and 120 mM. (c) TCA/acetone precipitation method. **(B)** Comparison of different methods of preparation of bovine milk whey in Indian *Sahiwal* cattle. (a) Acid precipitation at different pH. (b) Ultracentrifugation at 65,000×*g* for 2 h. (c) Ultracentrifugation followed by acid precipitation.

Supplementary Figure 2 Two-dimensional gel electrophoretogram of bovine milk whey in Indian *Sahiwal* cattle.

Supplementary Figure 3 Animal-to-animal proteome SDS profile of 18 different animals shown on two individual gels **(A,B)**. Ten best sharing proteome profiles were selected for the determination of deep whey proteome.

Supplementary Figure 4 All the different Gene Ontological Information determined for the whey proteome data shown in **(A–J)**. **(A)** ngLog mapped total proteome. **(B)** Functional annotation terms specified. **(C)** Complete cellular component counts. **(D)** The total molecular function counts. **(E)** The total biological function counts. **(F)** Full KEGG pathway annotation. **(G)** Total SMART term counts. **(H)** Total InterPro terms counts. **(I)** Tissue-specific proteins determined. **(J)** Total reactome term counts.

Supplementary Figure 5 Fuzzy C-means clustering of proteins identified with the metal ion binding ability.

Supplementary Table 1 Final complete table for identifications of total whey proteins in Trans Proteomics Pipeline (TPP) using Comet and Tandem search engine through *in-gel* and *in-solution* digestion.

Supplementary Table 2 Final complete table for identifications of total whey proteins in ProteinScape using Mascot search engine through *in-gel* and *in-solution* digestion.

Supplementary Table 3 Total number of chromosome-associated spectra counts, total proteins mapped, and enrichment values.

Supplementary Table 4 ngLOC-based classification of whey proteome in respective cellular organelle classification.

Supplementary Table 5 DAVID-based classification of all the proteins mapped in whey proteome.
