# Supplementary figures and images for: High-Resolution Mass Spectrometer–Based Ultra-Deep Profile of Milk Whey Proteome in Indian Zebu (Sahiwal) Cattle
Source: Front Nutr. 2020 Sep 11;7:150. doi: 10.3389/fnut.2020.00150 (PMC7533583; doi:10.3389/fnut.2020.00150)

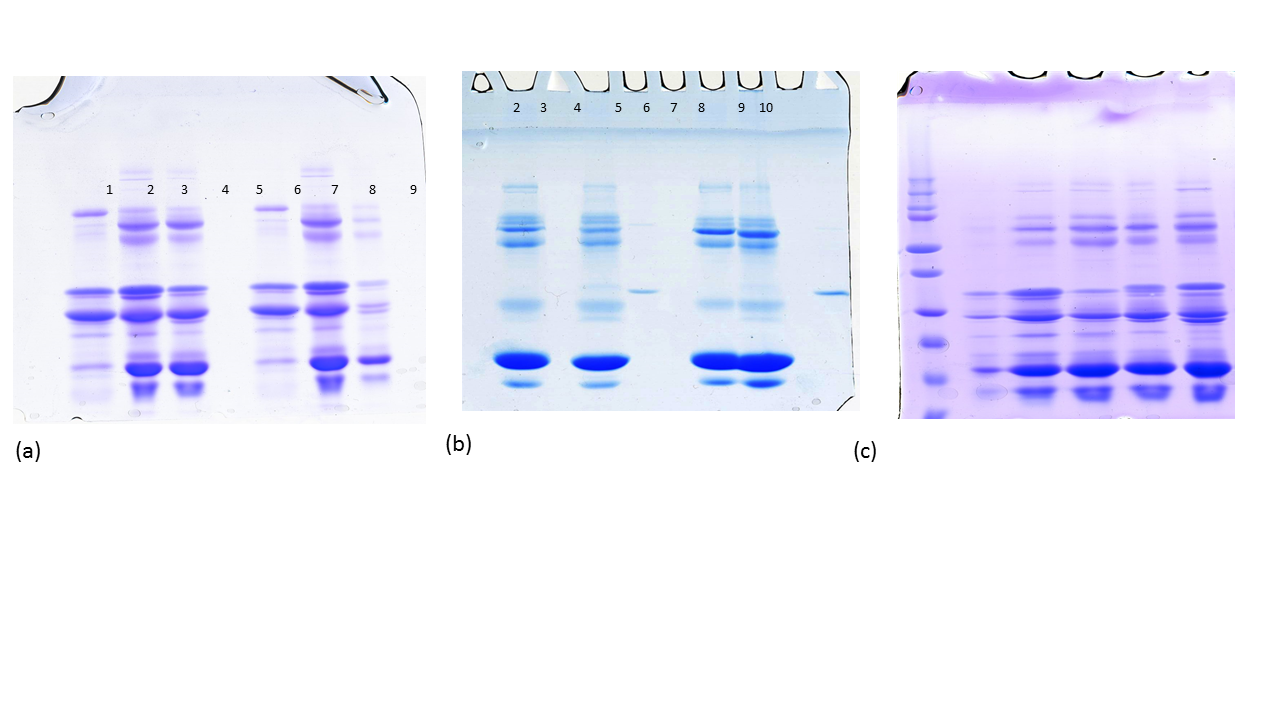

Supplement: Supplementary Figure 1 — (A) Comparison of SDS-PAGE patterns of milk whey samples for optimization of different extraction procedures. 1D image of milk whey protein extraction procedures followed for optimization. (a) Ultracentrifugation followed by acetone precipitation. (b) CaCl2 precipitation method at different concentrations: 60, 90, and 120 mM. (c) TCA/acetone precipitation method. (B) Comparison of different methods of preparation of bovine milk whey in Indian Sahiwal cattle. (a) Acid precipitation at different pH. (b) Ultracentrifugation at 65,000×g for 2 h. (c) Ultracentrifugation followed by acid precipitation. [file Image_1.tif]

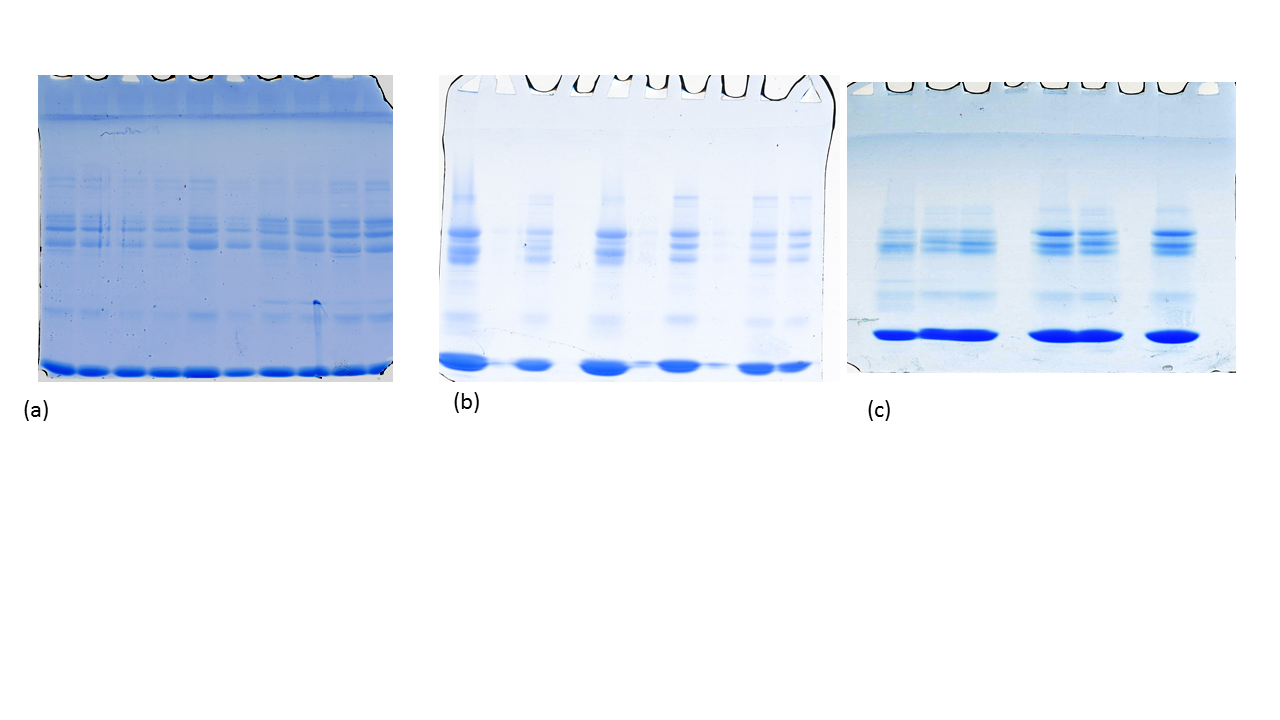

Supplement: Supplementary Figure 2 — Two-dimensional gel electrophoretogram of bovine milk whey in Indian Sahiwal cattle. [file Image_2.tif]

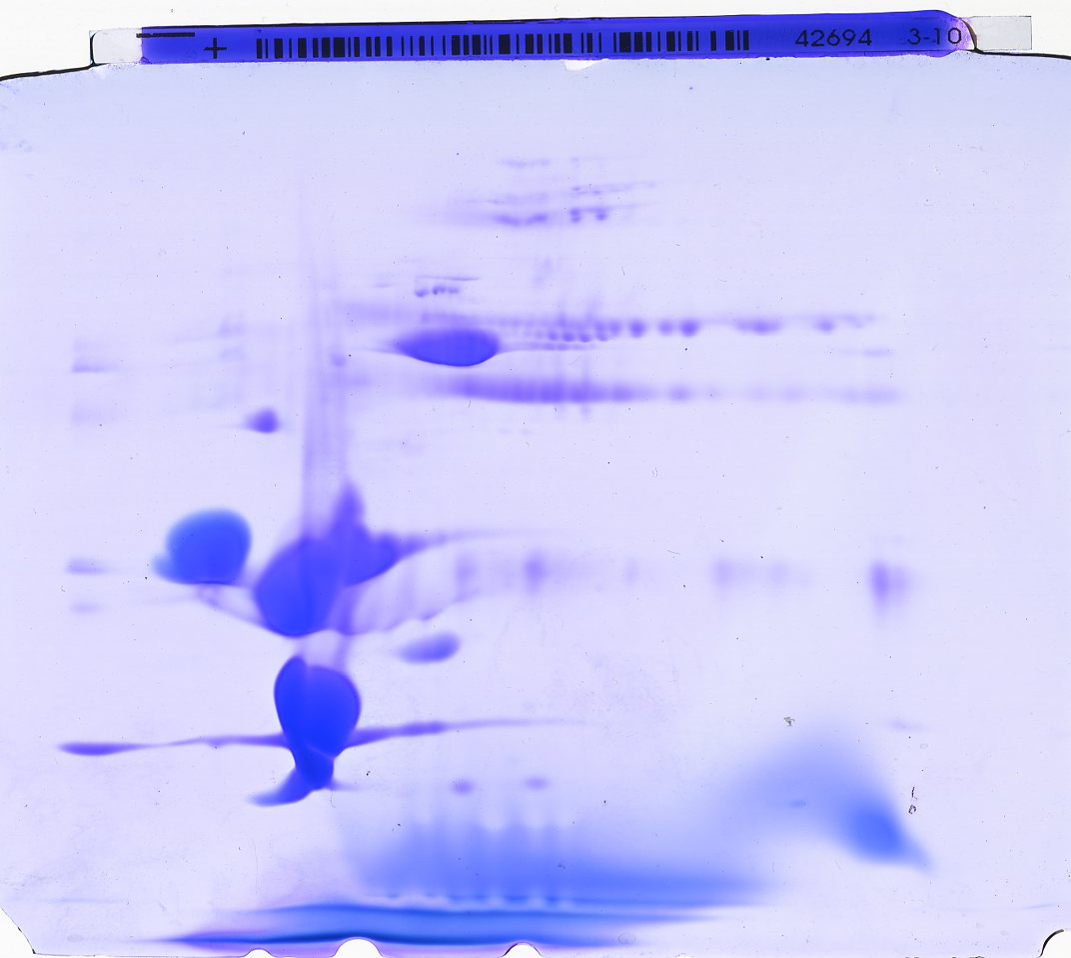

Supplement: Supplementary Figure 3 — Animal-to-animal proteome SDS profile of 18 different animals shown on two individual gels (A,B). Ten best sharing proteome profiles were selected for the determination of deep whey proteome. [file Image_3.TIF]

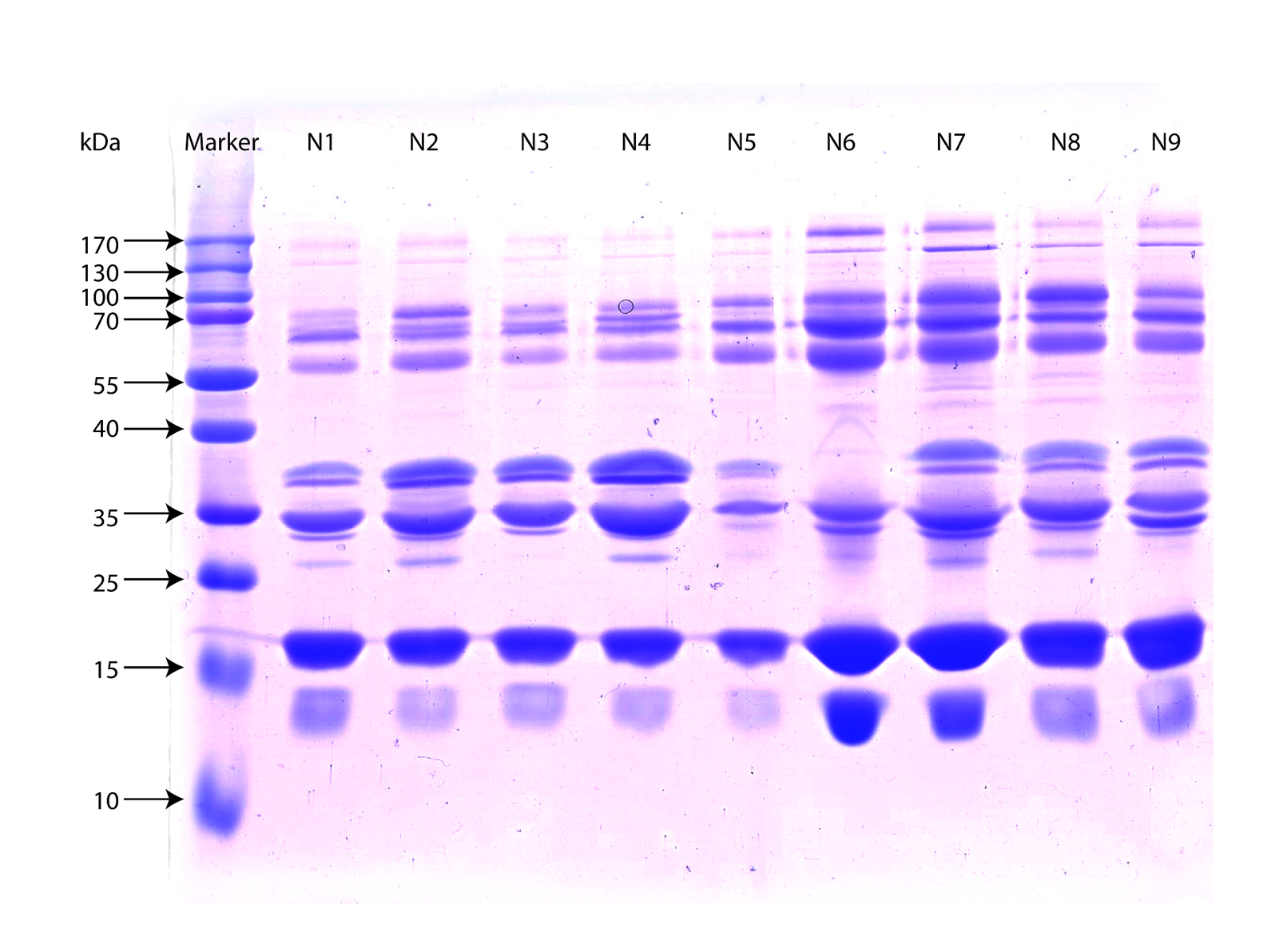

Supplement: Supplementary Figure 4 — All the different Gene Ontological Information determined for the whey proteome data shown in (A–J). (A) ngLog mapped total proteome. (B) Functional annotation terms specified. (C) Complete cellular component counts. (D) The total molecular function counts. (E) The total biological function counts. (F) Full KEGG pathway annotation. (G) Total SMART term counts. (H) Total InterPro terms counts. (I) Tissue-specific proteins determined. (J) Total reactome term counts. [file Image_4.TIF]

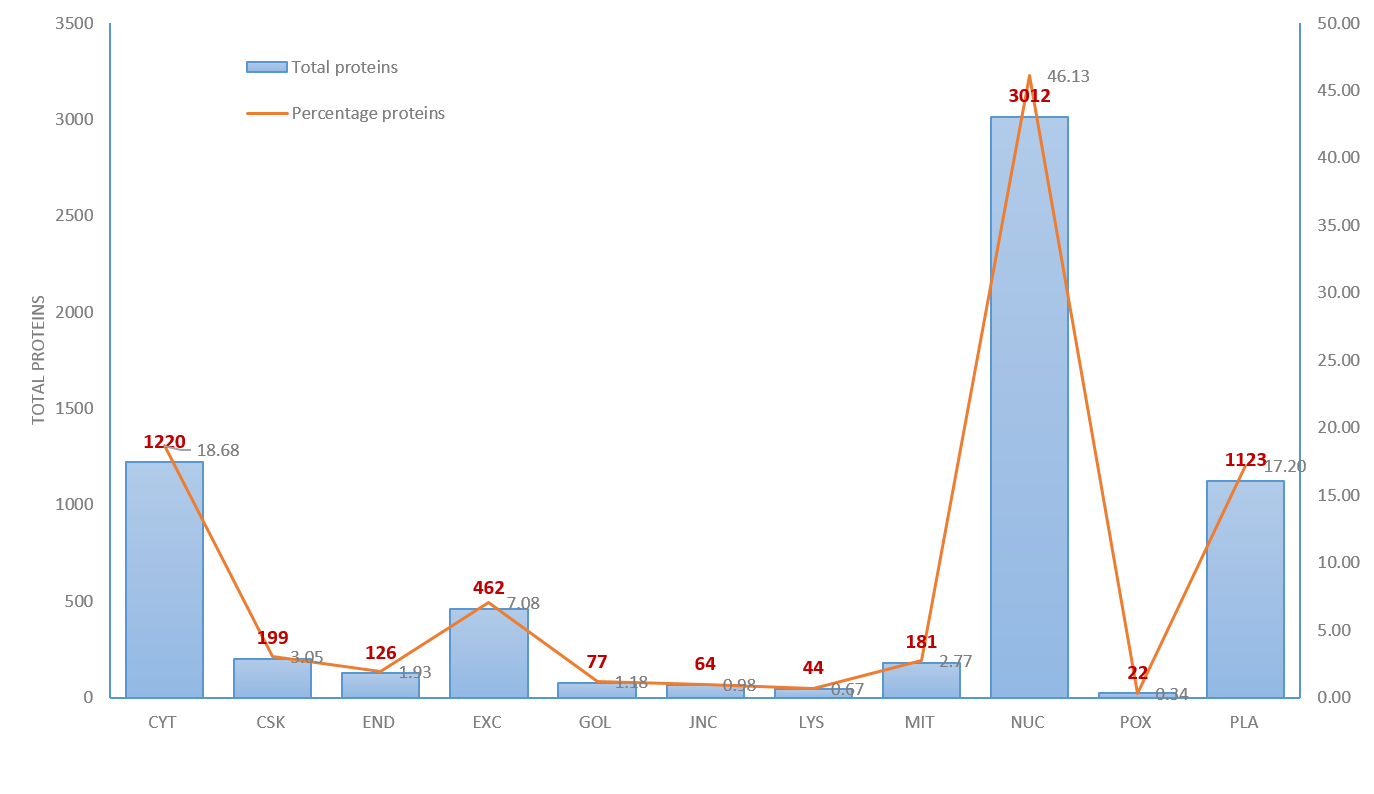

Supplement: Supplementary file 5 [file Image_5.TIF]

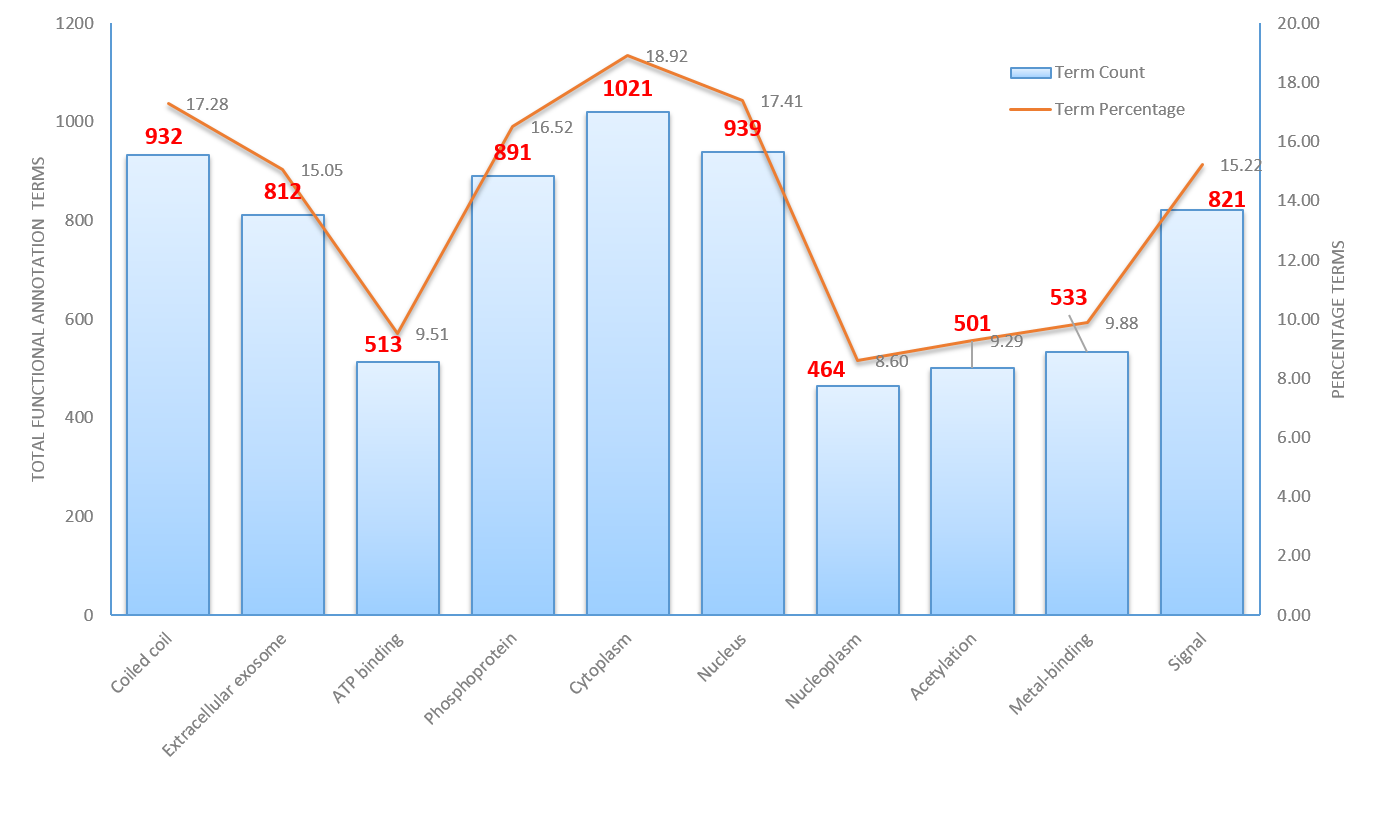

Supplement: Supplementary file 6 [file Image_6.TIF]

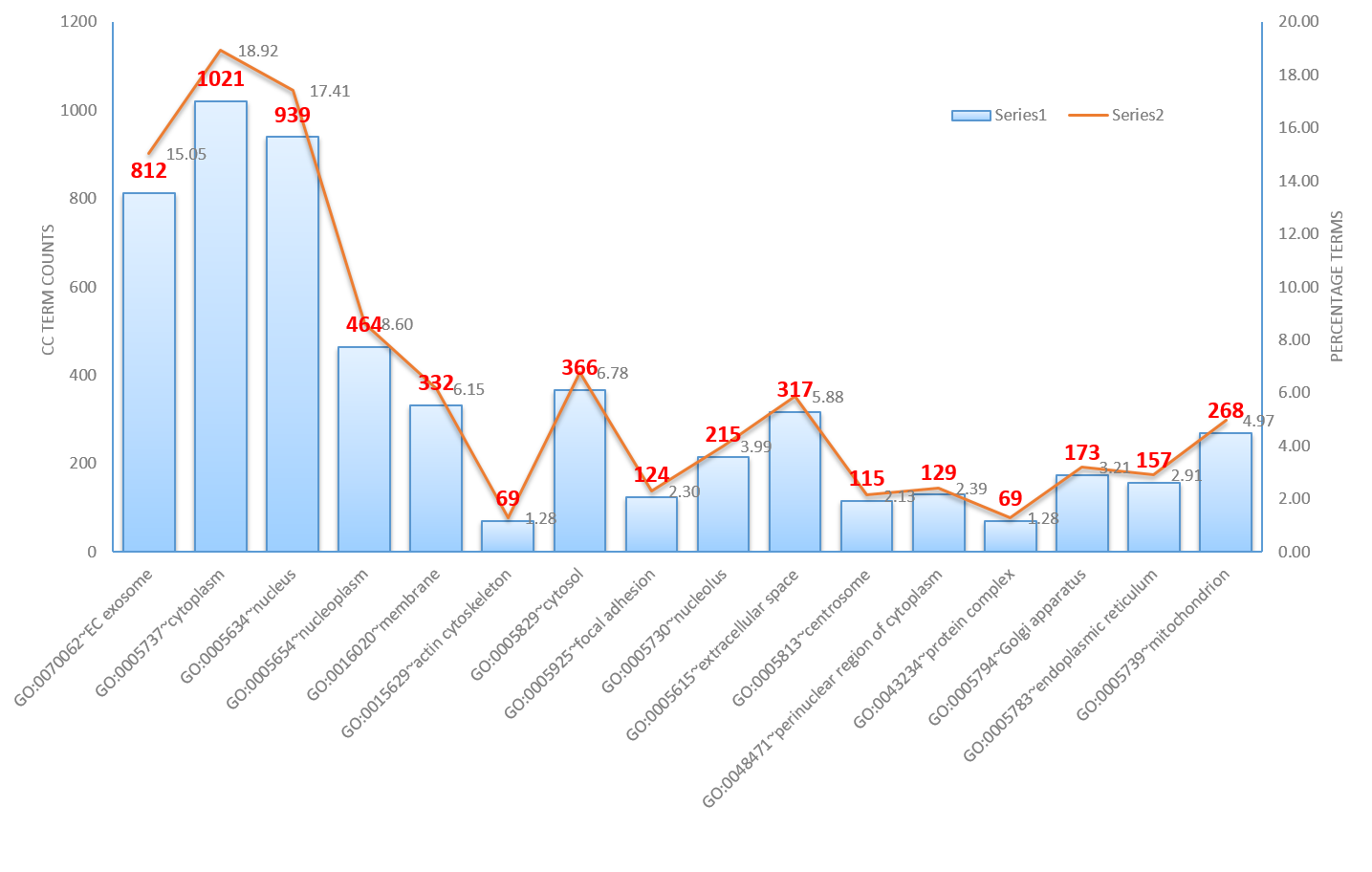

Supplement: Supplementary file 7 [file Image_7.TIF]

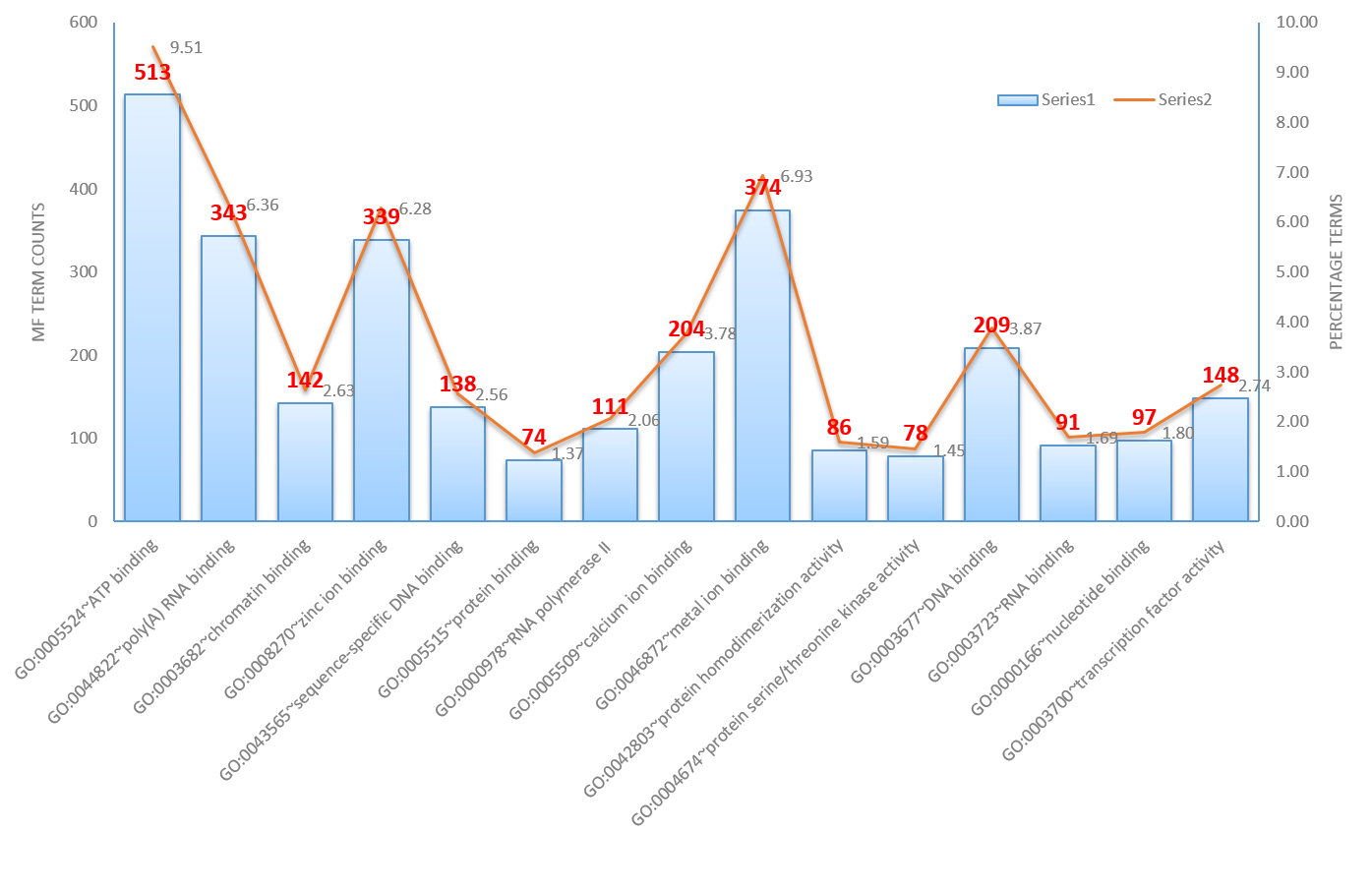

Supplement: Supplementary file 8 [file Image_8.TIF]

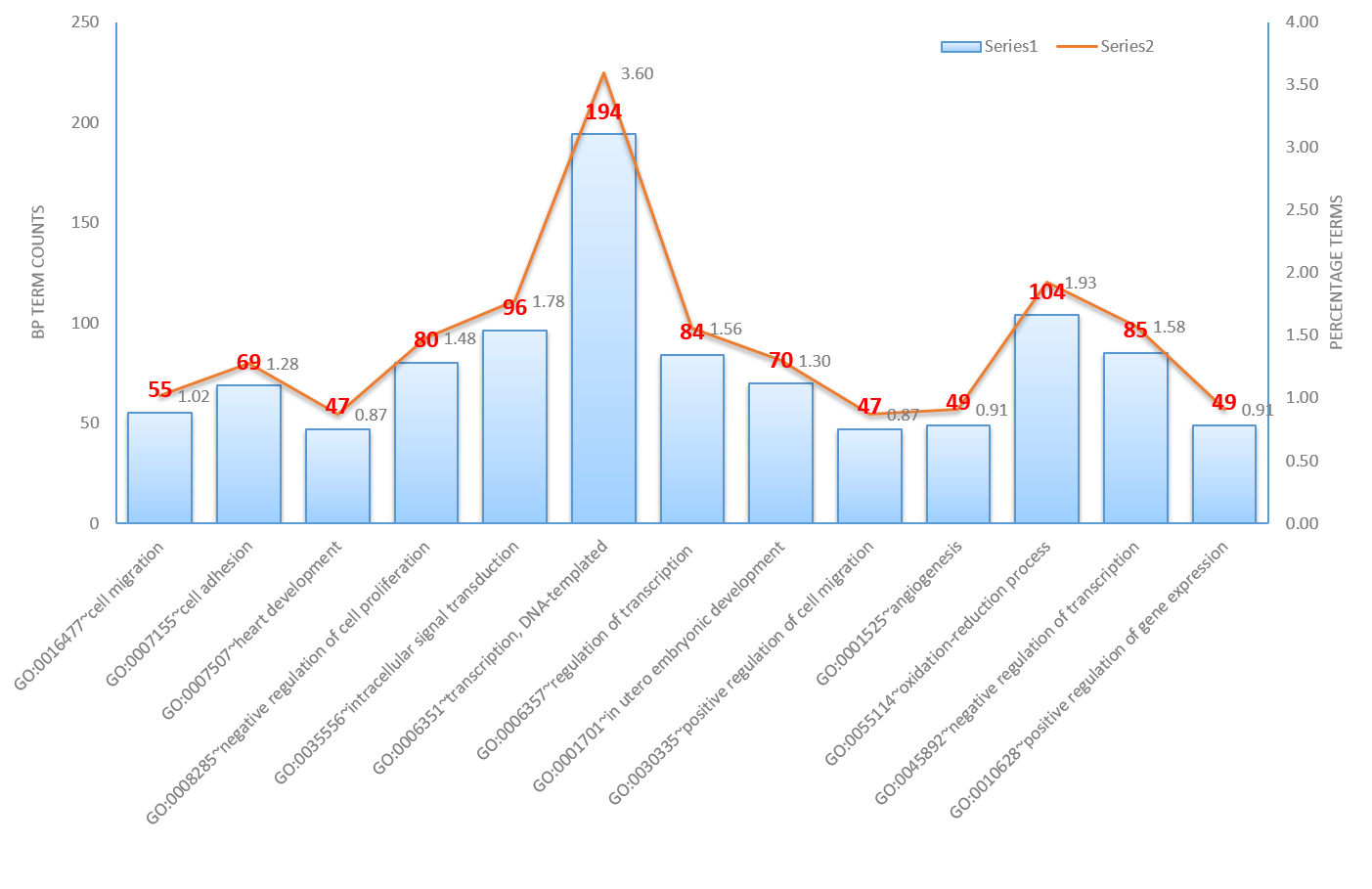

Supplement: Supplementary file 9 [file Image_9.TIF]

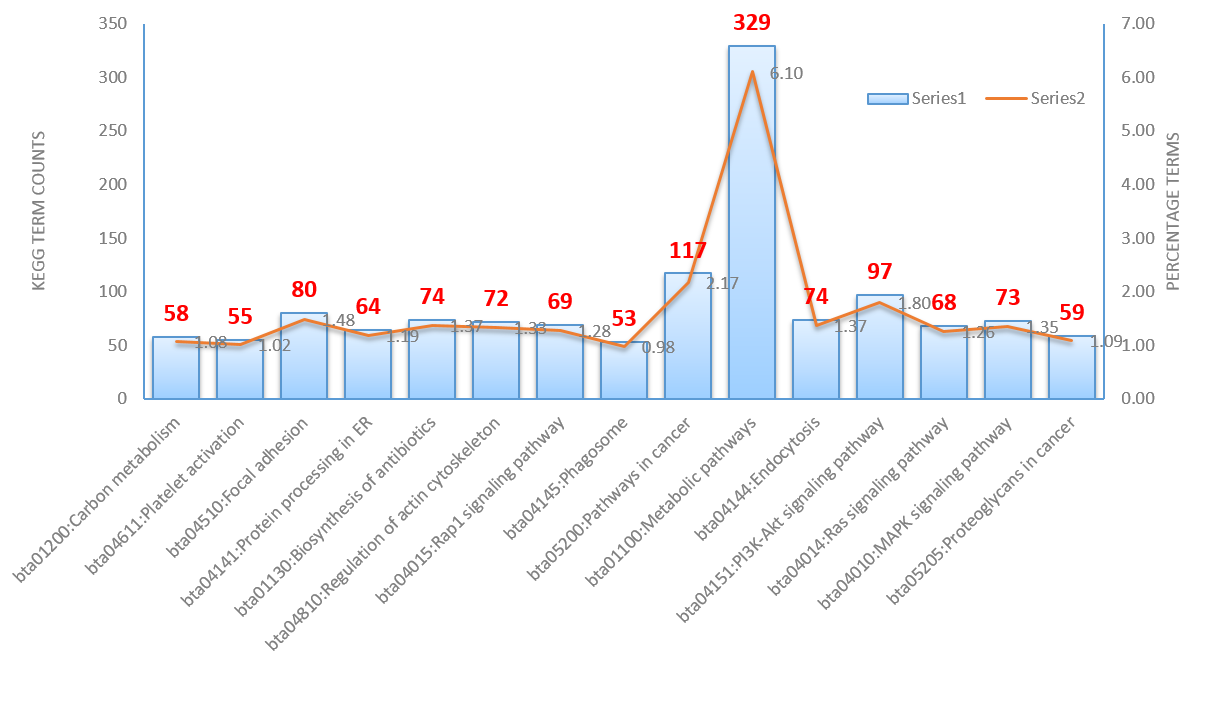

Supplement: Supplementary file 10 [file Image_10.TIF]

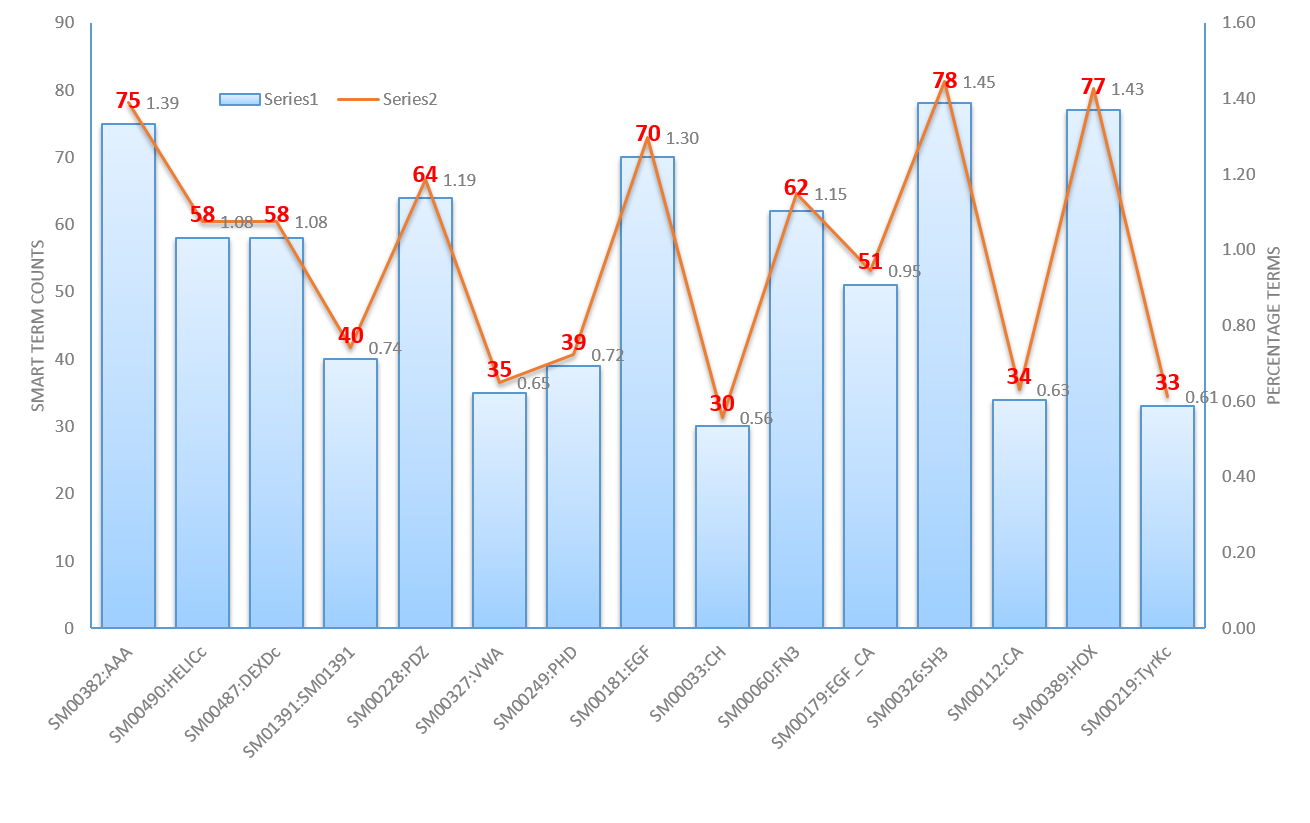

Supplement: Supplementary file 11 [file Image_11.TIF]

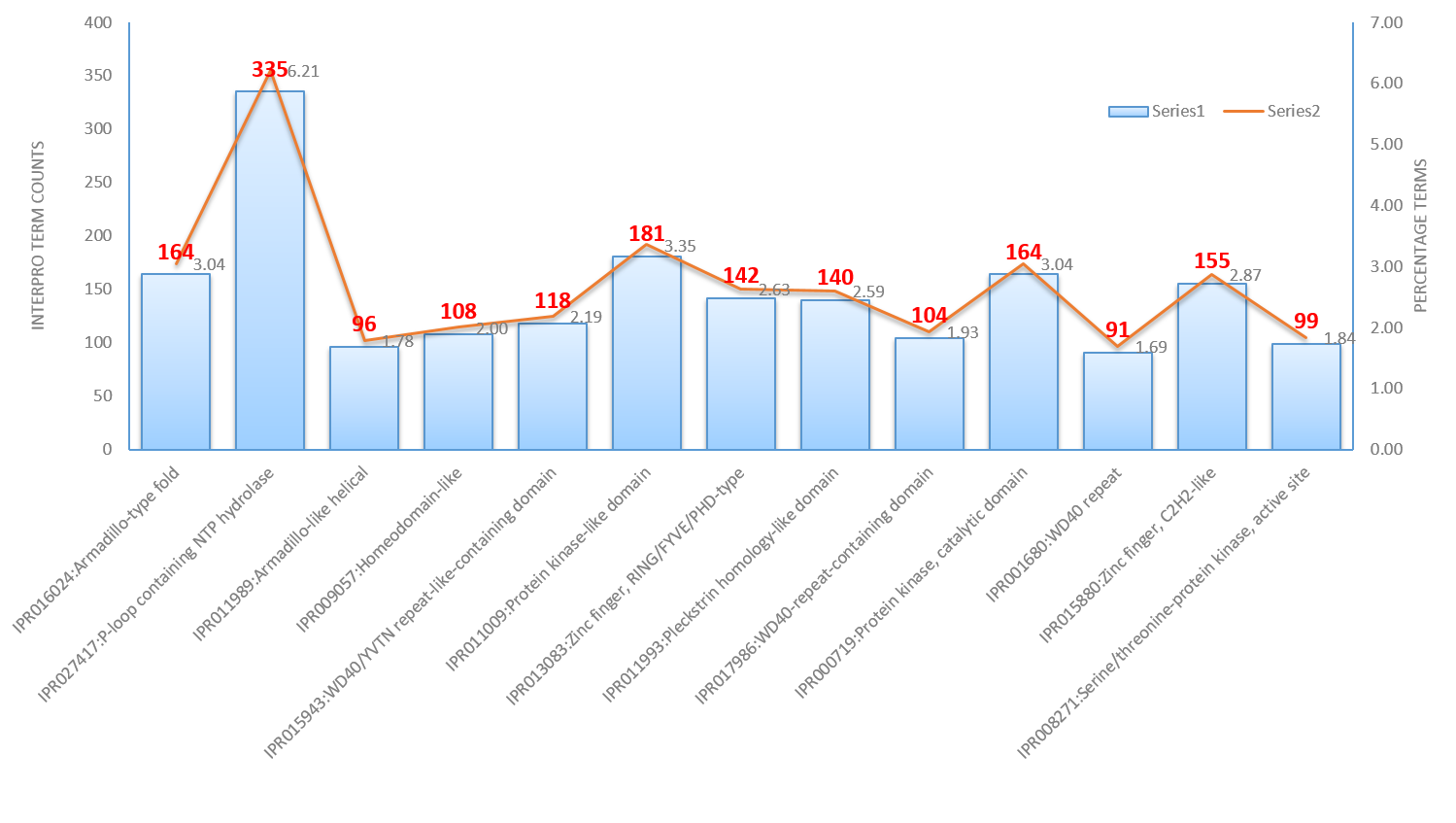

Supplement: Supplementary file 12 [file Image_12.TIF]

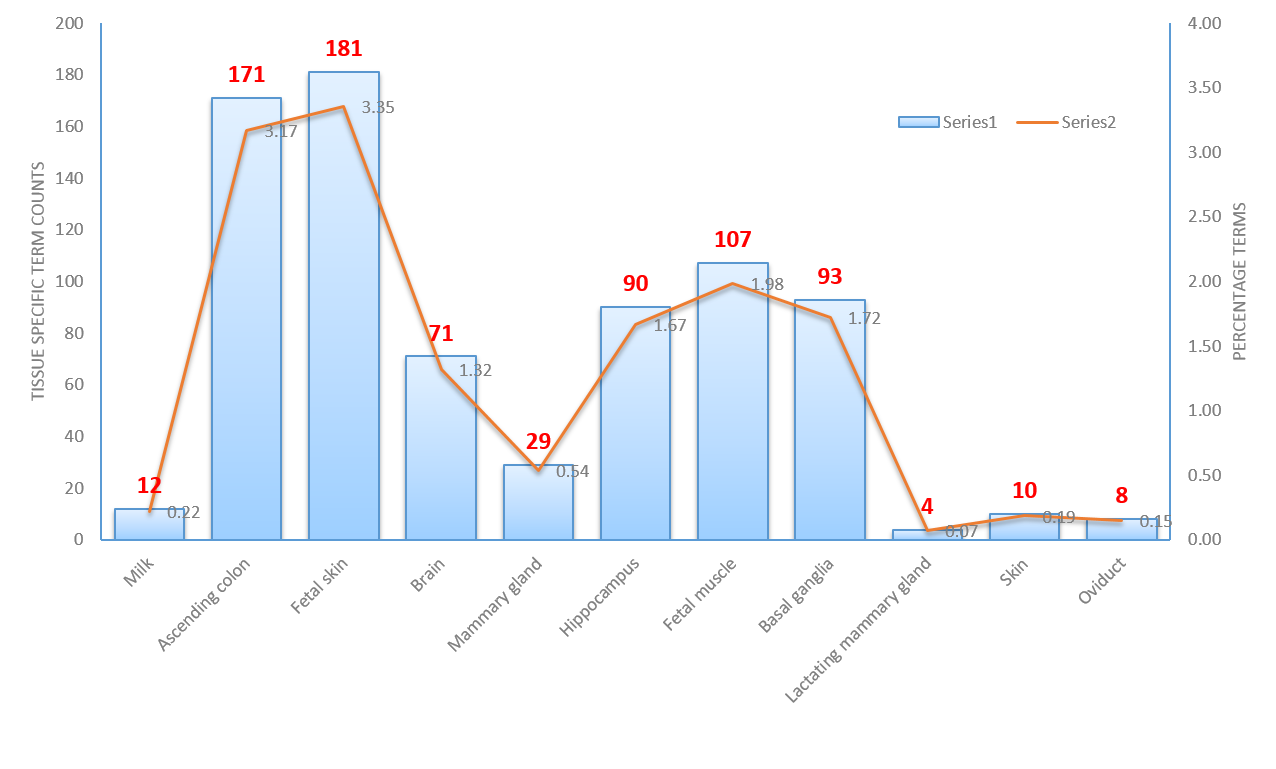

Supplement: Supplementary file 13 [file Image_13.TIF]

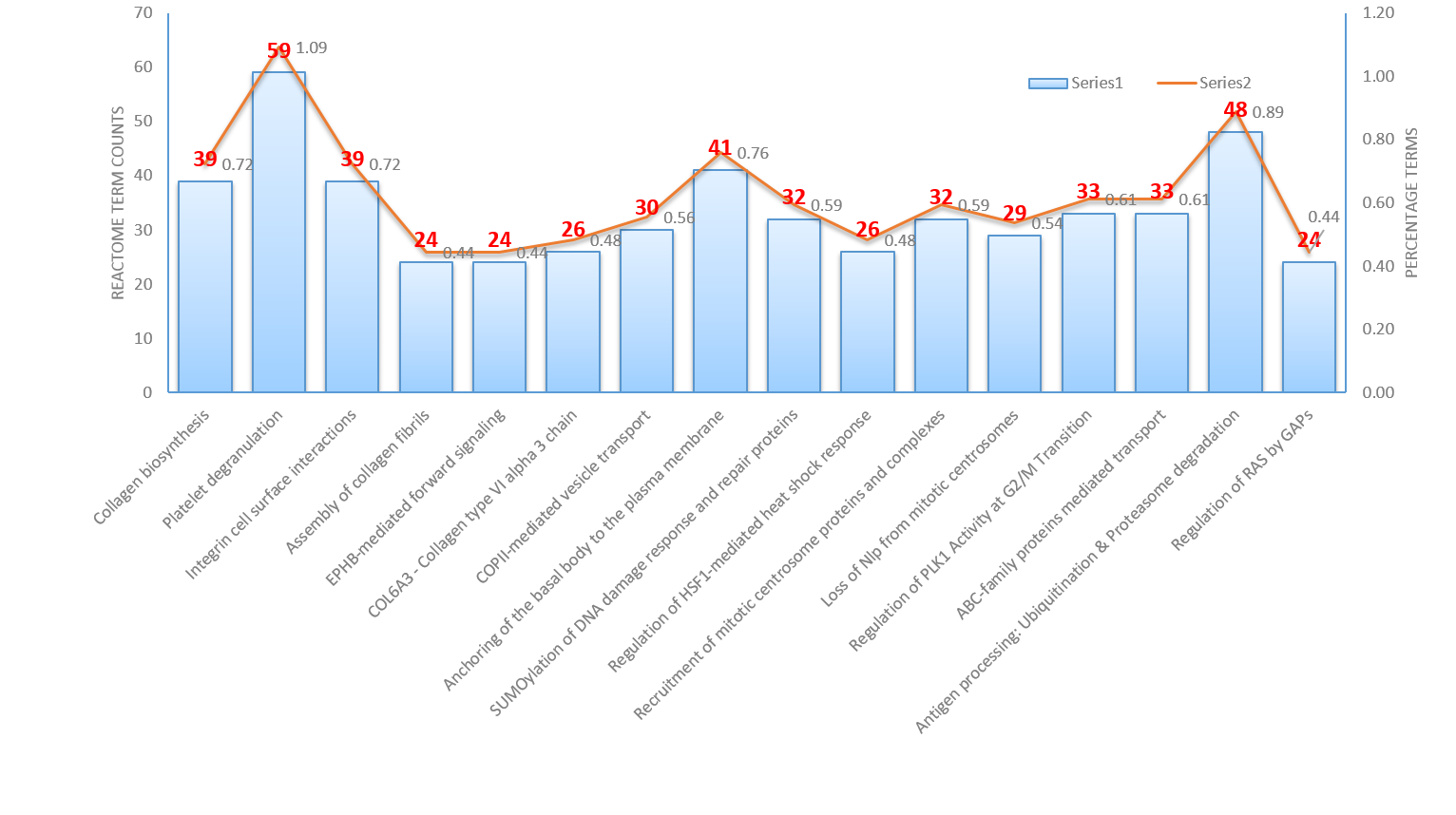

Supplement: Supplementary file 14 [file Image_14.TIF]

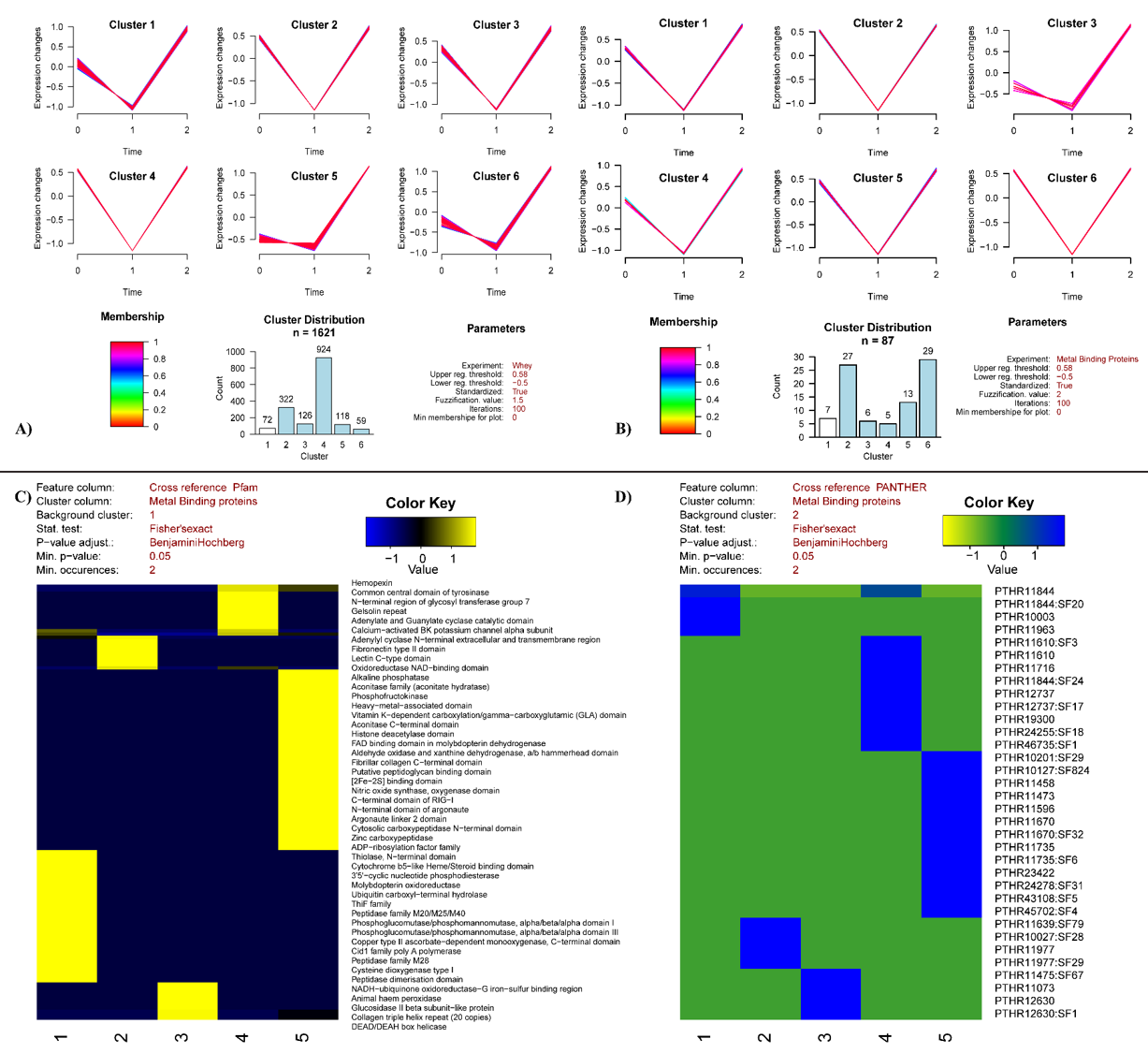

Supplement: Supplementary Figure 5 — Fuzzy C-means clustering of proteins identified with the metal ion binding ability. [file Image_15.TIF]
